# Sodium oligomannate alters gut microbiota, reduces cerebral amyloidosis and reactive microglia in a sex-specific manner

**DOI:** 10.1186/s13024-023-00700-w

**Published:** 2024-02-17

**Authors:** Megan E. Bosch, Hemraj B. Dodiya, Julia Michalkiewicz, Choonghee Lee, Shabana M. Shaik, Ian Q. Weigle, Can Zhang, Jack Osborn, Aishwarya Nambiar, Priyam Patel, Samira Parhizkar, Xiaoqiong Zhang, Marie L. Laury, Prasenjit Mondal, Ashley Gomm, Matthew John Schipma, Dania Mallah, Oleg Butovsky, Eugene B. Chang, Rudolph E. Tanzi, Jack A. Gilbert, David M. Holtzman, Sangram S. Sisodia

**Affiliations:** 1 Department of Neurology, Hope Center for Neurological Disorders, Knight Alzheimer’s Disease Research Center, Washington University in St. Louis, St. Louis, USA; 2 Department of Neurobiology, University of Chicago, Chicago, USA; 3 Genetics and Aging Research Unit, McCance Center for Brain Health, MassGeneral Institute for Neurodegenerative Disease, Department of Neurology, Massachusetts General Hospital and Harvard Medical School, Boston, MA USA; 4 Center for Genetic Medicine, Northwestern University, Chicago, USA; 5 Genome Technology Access Center, Washington University in St. Louis, St. Louis, USA; 6 Ann Romney Center for Neurologic Diseases, Department of Neurology, Brigham and Women’s Hospital, Harvard Medical School, Boston, MA USA; 7 Department Medicine, Section of Gastroenterology, Hepatology, and Nutrition, The University of Chicago, Chicago, USA; 8 Department of Pediatrics and Scripps Institution of Oceanography, UCSD, San Diego, USA

**Keywords:** Alzheimer’s disease, Microbiome, Sodium oligomannate, Microglia, Neuroinflammation

## Abstract

It has recently become well-established that there is a connection between Alzheimer’s disease pathology and gut microbiome dysbiosis. We have previously demonstrated that antibiotic-mediated gut microbiota perturbations lead to attenuation of Aβ deposition, phosphorylated tau accumulation, and disease-associated glial cell phenotypes in a sex-dependent manner. In this regard, we were intrigued by the finding that a marine-derived oligosaccharide, GV-971, was reported to alter gut microbiota and reduce Aβ amyloidosis in the 5XFAD mouse model that were treated at a point when Aβ burden was near plateau levels. Utilizing comparable methodologies, but with distinct technical and temporal features, we now report on the impact of GV-971 on gut microbiota, Aβ amyloidosis and microglial phenotypes in the APPPS1-21 model, studies performed at the University of Chicago, and independently in the 5X FAD model, studies performed at Washington University, St. Louis.

**Methods** To comprehensively characterize the effects of GV-971 on the microbiota-microglia-amyloid axis, we conducted two separate investigations at independent institutions. There was no coordination of the experimental design or execution between the two laboratories. Indeed, the two laboratories were not aware of each other’s experiments until the studies were completed. Male and female APPPS1-21 mice were treated daily with 40, 80, or 160 mg/kg of GV-971 from 8, when Aβ burden was detectable upto 12 weeks of age when Aβ burden was near maximal levels. In parallel, and to corroborate existing published studies and further investigate sex-related differences, male and female 5XFAD mice were treated daily with 100 mg/kg of GV-971 from 7 to 9 months of age when Aβ burden was near peak levels. Subsequently, the two laboratories independently assessed amyloid-β deposition, metagenomic, and neuroinflammatory profiles. Finally, studies were initiated at the University of Chicago to evaluate the metabolites in cecal tissue from vehicle and GV-971-treated 5XFAD mice.

**Results** These studies showed that independent of the procedural differences (dosage, timing and duration of treatment) between the two laboratories, cerebral amyloidosis was reduced primarily in male mice, independent of strain. We also observed sex-specific microbiota differences following GV-971 treatment. Interestingly, GV-971 significantly altered multiple overlapping bacterial species at both institutions. Moreover, we discovered that GV-971 significantly impacted microbiome metabolism, particularly by elevating amino acid production and influencing the tryptophan pathway. The metagenomics and metabolomics changes correspond with notable reductions in peripheral pro-inflammatory cytokine and chemokine profiles. Furthermore, GV-971 treatment dampened astrocyte and microglia activation, significantly decreasing plaque-associated reactive microglia while concurrently increasing homeostatic microglia only in male mice. Bulk RNAseq analysis unveiled sex-specific changes in cerebral cortex transcriptome profiles, but most importantly, the transcriptome changes in the GV-971-treated male group revealed the involvement of microglia and inflammatory responses.

**Conclusions** In conclusion, these studies demonstrate the connection between the gut microbiome, neuroinflammation, and Alzheimer’s disease pathology while highlighting the potential therapeutic effect of GV-971. GV-971 targets the microbiota-microglia-amyloid axis, leading to the lowering of plaque pathology and neuroinflammatory signatures in a sex-dependent manner when given at the onset of Aβ deposition or when given after Aβ deposition is already at higher levels.

**Supplementary Information:**

The online version contains supplementary material available at 10.1186/s13024-023-00700-w.

## Background

Alzheimer’s disease (AD) is a progressive neurodegenerative disorder that is pathologically characterized by the presence of abundant amyloid plaques, composed of amyloid-β (Aβ) peptides, and neurofibrillary tangles, composed of hyperphosphorylated/aggregated forms of tau in the cortex and hippocampus of affected individuals [1–3]. In addition to these pathological hallmarks, neuroinflammation accompanied by microgliosis, reactive astrogliosis, and upregulated proinflammatory cytokines are considered significant aspects of AD pathophysiology [4–6]. Despite advances in understanding the molecular and cellular mechanism(s) that drive disease, there is a paucity of therapeutics that can affect disease progression.

Gut microbiota have gained significant attention in the field of neuroscience. Multiple studies have provided evidence for a causal role of microbiota in autism, anxiety, depression, schizophrenia, Parkinson’s disease, multiple system atrophy, and AD (as reviewed in [7]). In patients with AD [8–13] and several transgenic mouse models of Aβ amyloidosis and tau accumulation, differences in gut microbiota composition compared with controls, have been documented indicating a connection between gut microbiota and Alzheimer’s pathophysiology [14–18]. In this regard, we and others have demonstrated that antibiotic (ABX)-mediated gut microbiota perturbations reduce Aβ deposition and tau accumulation in independent mouse models of Aβ amyloidosis and tau [14–18]. Surprisingly, the reduction in Aβ amyloidosis and suppression of cerebral neuroinflammation is only observed in male mice [19–21]. In addition, Aβ amyloidosis is significantly reduced in APPPS1-21 and 5xFAD mouse models raised in germ-free (GF) conditions, thus strengthening the role of gut microbiota in Aβ amyloidosis [22, 23]. Most importantly, when ABX-treated or GF mice were re-colonized with AD mouse gut microbiota, the levels of amyloidosis were restored, thus establishing a causal link between gut microbiota, amyloidosis, and tau pathology [20, 22, 23]. Specifically, Mezo and colleagues demonstrated that in GF 5xFAD mice, hippocampal microglial uptake of Aβ deposits was enhanced, resulting in decreased Aβ burden and rescue of neuronal loss and behavior improvements. We have shown that ABX-mediated gut microbiota perturbations failed to reduce Aβ amyloidosis in mice fed with a colony-stimulating factor-1 receptor (CSF-1R) antagonist that depletes microglia [20], indicating an essential role for microglia in the microbiota-amyloid axis.

Despite the wealth of investigations and mechanistic insights into AD pathophysiology, there is currently a paucity of therapeutics that can either treat or alter the progression of the disease. However, GV-971, a marine-derived oligosaccharide that was developed and extensively studied [17, 24] by the company Shanghai Green Valley Pharmaceuticals (GV), was shown to improve spatial learning and memory in mouse models of AD, inhibited neuroinflammation by markedly altering the composition of gut microbiota and in a concomitant reduction in Aβ plaque levels [17]. Moreover, GV-971 improved cognition with sustained improvement over 36 weeks in patients with mild to moderate AD dementia in a randomized, double-blind, placebo-controlled, multicenter phase III trial (NCT02293915) conducted in China [24].

The studies by Wang and colleagues [17] show that GV-971 reconditioned gut microbiota dysbiosis, suppressed neuroinflammation, reduced Aβ burden, and lead to a reversal of cognitive impairment. To validate and extend these findings focusing on microglial function and neuroinflammatory profiles, we employed APPPS1-21 and 5XFAD transgenic models of Aβ amyloidosis [25]. We tested the effects of GV-971 in a dose-, and sex-dependent manner in APPPS1-21 mice, while 5XFAD mice were treated with a single dose in both sexes. In APPPS1-21 mice, we orally gavaged 2-month-old male and female APPPS1-21 mice, a time point at the onset of cerebral Aβ pathology until the age of 3 months, while 5XFAD male and female mice were gavaged orally at 7 months of age, a time point with extensive amyloidosis, until the age of 9 months, with GV-971. In both settings, we observed a significant reduction in amyloidosis that occurred in a sex-dependent manner; male mice showed the most profound decrease in amyloidosis. GV-971 also altered neuroinflammatory profiles, specifically reduced plaque-localized disease-associated microglia, increased homeostatic microglia, and reduced reactive astrocytes only in male mice. RNAseq analysis further revealed alterations in microglia phagocytosis activity, complement system-related inflammatory response, and other inflammatory responses. None of these changes were observed in female groups. Finally, GV-971 treatment resulted in sex-specific changes in the gut microbiota, altering the β diversity as well as multiple overlapping bacterial species changes in the APPPS1-21 and 5XFAD models. These changes correlated with alterations in microbial metabolism and reductions in peripheral inflammation. Collectively, our combined efforts performed in different laboratories with differing mouse lines and without any coordination in experimental design, execution, or initial knowledge of each other’s experiments lead to the conclusion that GV-971 targets the microbiota-microglia-amyloid axis to alleviate neuroinflammation and AD plaque pathogenesis in a sex-dependent fashion.

## Methods

### Animal housing, handling and GV971 treatment

#### U. Chicago APPPS1-21

Heterozygous male and female APP_SWE_PS1_L166P_ (APPPS1-21) mice were maintained on a C57BL/6J background. Heterozygous pups were generated using breeding pairs of (APPPS1-21 Tg male) or (APPPS1-21 Tg female) or (APPPS1-21 dTg x C57BL/6J nonTg). Mice were housed in the University of Chicago Animal Resources Center (ARC) facility under stable housing conditions with a sterile micro-isolator cage (specific-pathogen-free SPF condition). Mice received ad libitum food (Teklad Global, #2918) and water. All experimental procedures were performed following the approved Animal Care and Use Protocols (ACUP) by the Institutional Animal Care and Use Committee (IACUC) of the University of Chicago.

Male and female APPPS1-21 mice were assigned to either vehicle or GV-971 groups in a staggered but randomized manner. A stock solution of three different dosages of GV-971 (40 mg/kg, 80 mg/kg, and 160 mg/kg) was prepared every week using autoclaved ARC drinking water. Using a 24-gauge 1-inch gavage needle (1.25 mm ball), we orally gavaged 8-week-old APPPS1-21 mice with 200 μl of GV-971 (40 mg/kg, 80 mg/kg, 160 mg/kg) every day for one month. Daily weekday/weekend gavages were performed by JM, ensuring that any handling-related distress was avoided in all mice. Also, to nullify any unavoidable handling-related stress effect, control groups of male and female mice received a daily gavage of 200 μl of autoclaved ARC drinking water. In this study, attention was given to mice developing any significant changes in health. Notably, there were no mice in the study that requires removal due to health issues. Mice were sacrificed at three months of age using the IACUC-approved euthanasia protocol.

#### WashU 5XFAD

Heterozygous male and female B6.Cg-Tg (APPSwFf1Lon, PSEN1*M146L*L286V) 6799Vas/Mmjax (5XFAD) mice [26]were purchased from Jackson Laboratory (MMRRC Strain #034840-JAX). All mice are on the C57BL/6 background. Heterozygous pups were generated using breeding pairs of (5XFAD Tg males x C57BL/6J nonTg females) or C57BL/6J nonTg males x 5XFAD females). Mice were housed under normal 12-h light/dark cycles under stable housing conditions with a sterile micro-isolator cage (specific-pathogen-free SPF condition). Mice received ad libitum food (PicoLab Rodent Diet 20; #5053) and water. All animal studies were approved by the Animals Studies Committee at Washington University School of Medicine in St. Louis.

Male and female 5XFAD mice were assigned to either vehicle or GV-971 groups in a randomized manner. A stock solution of GV-971 was prepared every week using autoclaved drinking water. Using an 18-gauge 2-inch gavage needle (1.25 mm ball), 7-month-old mice were orally gavaged every day with 100 mg/kg of GV-971 or vehicle for 2 months. Daily weekday/weekend gavages were performed by MEB, ensuring that any handling-related distress was avoided in all mice. To nullify any unavoidable handling-related stress, vehicle mice received a daily gavage of autoclaved drinking water. Mice were sacrificed at 9 months of age using IACUC-approved euthanasia protocol.

### Necropsy and tissue harvesting

#### U. Chicago APPPS1-21

Perfusion and tissue harvesting were carried out as described in [19]. On the day of sacrifice, mice were sedated using ketamine/xylazine as per the approved ACUP. The blood samples were collected by cardiac puncture using a 25-gauge needle and stored at 4^0^C, in sodium citrate buffer tubes (BD Vacutainer; #363083) prior to the centrifugation. Transcardial perfusion was performed using physiological pH cold saline through the left-ventricle and simultaneously clamping the descending aorta. Harvested brains were dissected into two hemispheres (one hemisphere was post-fixed with 4% paraformaldehyde and the other was frozen for RNA extractions and MSD biochemical analysis). Cecum was collected from the non-perfused lower body and stored at -80^0^C until the use. Plasma samples were separated by centrifugation at 2,000 rpm for 10 min at 4^0^C and stored at -80^0^C.

#### WashU 5XFAD

Perfusion and tissue harvesting was carried out as described in [27]. On the day of sacrifice, mice were sacrificed by intraperitoneal injection of pentobarbital (200 mg/kg). Blood samples were collected in EDTA-treated tubes before cardiac perfusion with 3 U/ml heparin in cold Dulbecco’s PBS. Blood samples were spun down (10 min, 2,000 × *g*, 4 °C), and blood plasma was collected. Transcardial perfusion was performed using physiological pH cold saline with 3 U/ml heparin. Brains were extracted and cut into two hemispheres. The left hemisphere was collected for immunostaining and immerse-fixed. The right hemisphere was dissected to isolate the hippocampus and cortex for biochemical analysis, and the tissue was kept at − 80 °C until analyzed. Fecal samples were collected using the clean catch method into 1.7 mL Eppendorf tubes and stored at -80^0^C until the use.

### Immunohistochemistry

#### U. Chicago APPPS1-21

Histology was performed as per the established procedure [19, 20, 28]. Briefly, extracted hemispheres were post-fixed using 4% paraformaldehyde for 24 h then, brains were transferred into 30% sucrose. Leica microtome (Leica; #SM210R,) was used to cut 40 µm thick coronal brain sections (from the beginning of the olfactory bulb till the end of hippocampal level sections) and stored in cryoprotectant solution. On the day of staining, a total of 6 level-matched sections at an equidistant interval of 480 µm were selected from each mouse for free-floating staining. Tissue slices were washed and blocked with serum-blocking solution for 1 h. Primary antibody specific for human amyloid (3D6, in house antibody, 1:10000) was used for 48 h incubation, and secondary antibody (Donkey-anti-mouse 488, 1:500, Thermofisher; # A-21202,) incubation for one hour. The sections were mounted on glass slides and then coverslipped using amount mounting media (ThermoFisher; # F4680,) prior to submission to the University of Chicago microscope core facility for full slide scanning. Immunofluorescence staining using primary antibodies (anti-amyloid (mouse-anti-Aβ, 3D6, 1:10000), homeostatic microglia (Rabbit-anti- P2ry12: Sigma-Aldrich; # HPA014518, 1:1000) and neurodegenerative-type microglia (Rat anti-mDectin-1: InvivoGen; # mabg-mdect, 1:250) markers) and respective secondary antibodies (Donkey-anti-mouse 488, Goat-anti-rat 555, Donkey-anti-rabbit 647) were performed to investigate the status of plaque-localized microglial cells. We used three level-matched sections at an equidistance of 960 µm to investigate the plaque-localized microglia status as described below.

#### WashU 5XFAD

Histology was performed as established in [27]. Briefly, extracted hemispheres were post-fixed using 4% paraformaldehyde for 48 h, then, brains were transferred into 30% sucrose. Leica microtome (Leica; #SM210R) was used to cut 30 µm thick coronal brain sections (from the beginning of the olfactory bulb till the end of hippocampal level sections) and stored in cryoprotectant solution. A total of 3 level-matched sections at an equidistant interval of 360 µm were selected from each mouse for free-floating staining. For Aβ plaque analysis, sections were washed three times in TBS for 5 min and blocked in 0.3% hydrogen peroxide for 10 min. After washing, sections were blocked in 3% milk in TBS with 0.25% Triton X-100 (TBSX) for 30 min. The sections were incubated in primary antibody (HJ3.4 biotinylated, anti-Aβ1–13, mouse monoclonal, 2 µg/ml generated in-house) overnight at 4 °C. Sections were incubated in ABC Elite solution (VectaStain; #PK-6100) for 1 h, prepared following the manufacturer’s instructions. Sections were developed in DAB solution (Sigma; #D5905), washed, and mounted on slides and cover slipped with Cytoseal 60 (Thermo Fisher Scientific; #8310). Immunofluorescence staining using primary antibodies (anti-amyloid (mouse-anti-amyloid, HJ3.4 biotinylated, anti-Aβ1–13, mouse monoclonal, 2 µg/ml, X-34 1;1000 Sigma; #SML1954), homeostatic microglia (Rabbit-anti- P2ry12: Biolegend; #S16007D, 1:100) and neurodegenerative-type microglia (Rat anti-mDectin-1(Clec7a): InvivoGen; #mabg-mdect, 1:100) markers), general microglia marker (Iba-1 Wako; 011–27991, 1:1000), astrocyte activation (GFAP Abcam; #ab134436, 1:1000) and respective secondary antibodies (Donkey-anti-rabbit 488 (#711–545-152), Donkey-anti-rat 594 (#712–585-150), Donkey-anti-chicken 647 (#703–605-155), Jackson ImmunoResearch) incubations were performed to investigate the status of plaque-localized microglia and astrocytes. Lipofuscin was quenched with 1X TrueBlack (Biotium; 23007) and washed once in PBS. Sections were mounted and sealed in ProLong Gold anti-fade (Thermo Fisher Scientific;#P36930). We used three level-matched sections at an equidistance of 360 µm to investigate the plaque-localized microglia and astrocytes status as described below.

### Amyloid-β burden measures

#### U. Chicago APPPS1-21

Amyloid quantification was performed as published previously [19, 20, 28]. In brief, slides were scanned using a slide scanner under a magnification of 20x to prepare a 3D Z-stack of each slide by the microscope core facility. Threshold-based analysis for Aβ burden was performed on these images using Fiji Image-J. The sections were converted into 8-bit images, followed by a selection of cerebral cortex following tissue landmarks. An appropriate threshold number (based on the preliminary analysis avoiding floor/ceiling effects) was applied consistently to highlight the majority of amyloid plaques ensuring no artifacts were incorporated in the quantification. Fill holes and watershed plugins were applied. Particles were analyzed using 10-400pixel^2^ size and 0.4–1.00 circularity criteria for each section. Collated numbers were collected to calculate the mean burden and mean particle size and compared between groups using GraphPad Prism software (Prism 7, version 7.0e, 2018).

#### WashU 5XFAD

Images were obtained from an average of 3 sections per mouse for IHC and IF. For IHC stains, slides were scanned on the NanoZoomer 2.0-HT system (Hamamatsu Photonics) at 20x. Images were further processed using NDP viewing software (Hamamatsu Photonics) and Fiji software version 1.51 (National Institutes of Health). The sections were converted into 8-bit images followed by a selection of cerebral cortex and hippocampus using tissue landmarks. An appropriate threshold number (based on the preliminary analysis avoiding floor/ceiling effects) was applied consistently to highlight the majority of amyloid plaques ensuring no artifacts were incorporated in the quantification. Particles were analyzed and collated numbers were collected to calculate the mean amyloid burden between groups using GraphPad Prism software. For IF (Iba1 and GFAP) stains and slides were scanned on Leica Thunder imager 3D assay at 20x. Images were further processed using Fiji software as previously described.

### Microglia (Clec7a^+^/P2ry12^+^ plaque-localized microglia) quantification

#### U. Chicago APPPS1-21

Immunofluorescence slides stained with markers for 3D6 (FITC), Clec7a (Cy5), and P2ry12 (Cy3) were scanned using Leica SP8 laser scanning confocal microscope under 63x/1.4 UV oil objective. 3D-Z stacks, 0.35 µm step increments in the z-plane, were prepared for 10 plaques-containing microenvironments per case. The images were then imported into Fiji Image-J for individual channel separation, and maximum-intensity projections, followed by manual counts of Clec7a^+^ and P2ry12^+^ microglia numbers in each compressed image containing Aβ plaque. The average number of Clec7a^+^ microglia and P2ry12^+^ microglia per image (0.02mm^2^ area) was generated and plotted using GraphPad Prism (Prism 7, version 7.0e, 2018).

#### WashU 5XFAD

Immunofluorescence slides were stained with markers for Ibal-1 (Alexa 488), Clec7a or P2ry12 (Alexa 594), and HJ3.4 (Alexa 647) were scanned using Leica SP5 laser scanning confocal microscope under 20x objective. Three 3D-Z stacks, 2 µm step increments in the z-plane, were acquired from 3 cortical tissues per mouse, 9 images total. Quantification of confocal images for Iba1/plaque, Clec7a/ plaque, and P2ry12/plaque was performed on a semi-automated platform using MATLAB and Imaris 9.3.1 software to create surfaces of each stain. The Aβ plaque surfaces were then extended 3-15 μm around and the number of counter stain surfaces within the plaque perimeter was quantified and plotted using GraphPad Prism (Prism 9.4.1).

### Cerebral cortex RNA extraction

#### U. Chicago APPPS1-21

Total RNA was isolated from the dorsal cerebral cortex similar to our previous work [20]. Briefly, the dorsal cerebral cortex was homogenized with TRIzol reagent and RNA was extracted. The quality of the total RNA was evaluated using the Agilent Bioanalyzer. RNA-seq library preparations and illumine HiSeq4000 were performed by the University of Chicago Genomics Core facility similar to sequencing generated previously [20]. The data files were collected in FASTQ format for the bioinformatic analysis.

#### WashU 5XFAD

Total RNA was isolated from whole cerebral cortex using the Trizol method followed by RNeasy Mini Kit (QIAGEN; catalog no. 74104) and prepared cDNA with the High-Capacity RNA-to-cDNA kit (Applied Biosystems; #4388950) following the manufacturer’s instructions. cDNA was further purified using a QIAquick PCR purification kit following the manufacturer’s instructions (QIAGEN; #28104).

#### U. Chicago APPPS1-21 RNA-seq bioinformatics analysis

The quality of RNA reads, in FASTQ format, was evaluated using FastQC [29] and similar to our published work [19]. In brief, adapters were trimmed, and reads of poor quality or aligning to rRNA sequences were filtered using Trim Galore (http://www.bioinformatics.babraham.ac.uk/projects/trim_galore/). The later reads were then aligned to the mouse genome (mm10) using STAR [30]. Read counts for each gene were calculated using HTSeq-Counts [29] in conjunction with a gene annotation file for mm10 obtained from Ensembl (http://useast.ensembl.org/index.html). We generated a comprehensive quality control report using MultiQC [30]. DEseq2 [31] was used to determine differential expression. The cut-off for determining significant DEGs was a *P* < 0.01. GO analysis and identification of DEGs belonging to specific pathways were performed using Metascape [32]. We also utilized Cytoscape to analyze string networks and especially to investigate the immune pathway-related targets. Gene set enrichment analysis (GSEA) was also utilized to compare our cerebral cortex total RNAseq-generated transcriptome data with available online deposited gene enrichment datasets. Data generated from these different platforms were utilized to generate Figs. 7 and 8.

#### U. Chicago APPPS1-21 reverse transcription quantitative PCR

We used 1 μg of RNA for cDNA synthesis using the SuperScript IV VILO Master Mix with exDNase Enzyme (ThermoFisher; #11766050) according to the kit instructions. After synthesis, cDNA was diluted 40 times in Nuclease-free water (Invitrogen; #AM9938). Primers used for the detection of the signal were designed either by using established ones in previously published methods or using Primer BLAST tool (NCBI distribution) and checked for dimer formation in Multiple Primer Analyzer software (ThermoFisher). cDNA were subject to qPCR using PowerUp SYBR Green Master Mix (Applied Biosystems by Life Technologies; #A25742,). The volume of each reaction was 10 μL, which contained 5μL of PowerUP SYBR Green Master Mix (2x), 0.5 μL of forward and reverse primer mixture (in concentration of 5 μM), 0.4 μL of Nuclease-free water, and 4 μL of cDNA added to wells individually. The reactions were run on MicroAmp Fast Optical 96-Well Reaction Plate with Barcode (0.1 mL, Applied Biosystems by Life Technologies;# 4346906) with MicroAmp Optical Adhesive Film (Applied Biosystems by Life Technologies; 4311971,) on QuantStudio 3 Real-Time PCR System (ThermoFisher; #A28567,). Amplification was performed starting with 3 min hold at 95 °C to activate the enzyme. Next, the template was denaturated at 95 °C for 20 s, then annealed at 60 °C for 20 s, which was followed by extension and data acquisition at 72 °C for 20 s.). In brief, the data was normalized by evaluation of Ct mean of a housekeeping gene (Cyc1) for each sample. Cyc1 was the most stable housekeeping gene, hence only this gene was included in analysis. The expression levels were then calculated according to the ΔΔCt method. To assess the statistical differences between gene expressions two-way ANOVA was performed. A *p*-value of < 0.05 was considered statistically significant.

#### WashU 5XFAD Fluidigm qPCR

Gene expression analysis was performed using microarray in collaboration with the Genome Technology Access Core at Washington University. Using TaqMan probes, the relative gene expression was quantitatively measured using Fluidigm Biomark HD with integrated fluidic circuits. The data was normalized by evaluation of geometricCt mean of housekeeping genes (GAPDH and β-actin) for each sample. The expression levels were then calculated according to the ΔΔCt method. To assess the statistical differences between gene expressions a t-test was performed. A *p*-value of < 0.05 was considered statistically significant.

#### U. Chicago APPPS1-21brain protein extraction

To quantify soluble and insoluble Aβ levels, we chose the ventral half of each frozen brain. Briefly, the -80 °C stored frozen brains were dissected into dorsal and ventral halves on dry ice. The ventral halves were weighed and homogenized with 5X (w/v) volume tris-buffered saline (TBS) containing 1X halt protease inhibitor cocktail (Thermo Fisher Scientific) and 5 mM EDTA. After sonication, the homogenized samples were subject to ultracentifugation at 100,000xg for 60 min at 4 °C. The supernatant fraction was collected to detect TBS soluble Aβ levels. The remaining pellet fraction was further extracted in 10X (w/v) volume of 70% formic acid (FA) followed by homogenization. The homogenized samples were then subject to ultracentrifugation at 100,000xg for 60 min at 4 °C to collect the supernatant fraction that contains TBS-insoluble and FA soluble Aβ levels. The supernatants were frozen immediately and shipped on dry ice to the Harvard University for MesoScale Aβ analysis.

#### U. Chicago APPPS1-21 MesoScale Aβ analysis

Levels of Aβ peptides were analyzed through protocols previously reported [33]. Specifically, the assay was performed on an electrochemi-luminescence-based multi-array method using the Quickplex SQ 120 system from MSD Meso Scale Diagnostics LLC. The MesoScale Aβ 4G8 kits were utilized to detect Aβ peptides in a 96-well-based assay. First, 96-well plates were blocked with diluents provided by the manufacturer with shaking for 1 h at room temperature (RT). The experimental samples and MesoScale protein standards were resuspended in the manufacturer-supplied detection antibodies. The mixed solutions were placed on a shaker for 2 h at RT, followed by washing and adding of the reading buffer. The electrochemiluminescence signals were captured and signals were obtained for all samples and standard proteins, and the sample Aβ levels were analyzed using the MesoScale protein standards.

#### U. Chicago APPPS1-21Cecal bacterial DNA extraction and microbiome analysis

We used 50-100 mg of cecal content to extract microbial DNA. DNA extractions were performed using Qiagen DNeasy PowerSoil Pro kit following the manufacturer’s protocol (Qiagen; # 47,016) by IQW. Purified DNA was submitted to the Argonne National Laboratories for 16 s rRNA amplicon sequencing (Illumin MiSeq) under EGC supervision. For sequencing analysis, Earth Microbiome Project raw sequences were imported into Qiime2 [34] and analyses were performed by HBD. We used Dada2 to demultiplex [33]. Followed by quality control, sequences were aligned using mafft [35] and a phylogenetic tree was constructed using fasttree [36]. Sampling depth was rarefied at 9000sequences per sample for combined comparison irrespective of sexes and separately at 14400sequences per sample for male groups and 9000sequences per sample for female groups to maximize depth while prioritizing equal retention of samples across groups [37]. Alpha diversity and β diversity were calculated as per established protocols [38–40]. Taxonomy was compiled using the classify-sklearn plugin with greengenes Greengenes 13_8 99% OTUs pre-trained Naïve Bayes classifier [41–43]. ANCOM (analysis of comparison of microbiome) was performed using ANCOM plugin to evaluate differentially abundant taxa at species level (L7) between male groups and female groups.

#### WashU 5XFAD fecal bacterial DNA extraction and microbiome analysis

We used 20-50 mg of fecal content to extract microbial DNA. DNA extractions were performed using Qiagen DNeasy PowerSoil Pro kit following the manufacturer’s protocol (Qiagen; # 47016) by MEB. Purified DNA was submitted to the Washington University McDonnell Genome Institute under MLL supervision. Seven PCR amplicons representative of all nine 16S variable regions using the primers indicated in Table 1 were generated using the Fluidigm Access Array System. Reaction mixture components included 10X Fast Start High Fidelity buffer without MgCl, 25 mM MgCl, dimethyl sulfoxide, 10 mM PCR Grade Nucleotide Mix, 0.05 U/µL of 5U/µL FastStart High Fidelity Enzyme Blend, 20X Access Array Loading Reagent, 1 µL DNA, and molecular grade water. The BioMark HD system from Fluidigm was employed for PCR amplification. Reaction products were indexed with unique 10 base pair sequences via 7 rounds of PCR in order to combine each index sequence. 48 sample libraries were constructed via sample pooling and bead purification used for cleaning. Illumina MiSeq sequencer (2 × 150 base pair kit) was used for library sequencing. Amplification and sequencing were performed at the Genome Technology Access Center at the McDonnell Genome Institute at Washington University in St. Louis. Demultiplexed reads from the 7 amplicons were analyzed using the MVRSION pipeline [44] to generate a list of microbial species with their corresponding number of reads for each sample. Default parameters were employed for the MVRSION analysis in conjunction with the Silva 16S database and data was rarefied to a depth of 11,000 for the female group and 22,000 for male groups. Diversity analysis was run for the following sample groupings: sex and treatment. The taxonomic classification results of MVRSION were post-processed via QIIME for the core set of diversity analyses, including alpha and beta diversity, alpha rarefaction, and group significance. Additional alpha diversity analysis employed QIIME2.

**Table 1:** GV-971 significantly alters microbiome bacterial species in male mice treated with 160 mg/kg compared with vehicle

**Comparisons**		
**Male Ctrl vs Male 160 mg/kg GV- 971**	**Increased in GV-971**	**Decreased in GV-971**
	p__Actinobacteria;c__Actinobacteria; o__Bifidobacteriales;f__Bifidobacteriaceae; g__Bifidobacterium;s__pseudolongum	p__Actinobacteria;c__Actinobacteria; o__Actinomycetales;f__Corynebacteriaceae; g__Corynebacterium;__
	p__Actinobacteria;c__Coriobacteriia; o__Coriobacteriales;f__Coriobacteriaceae; g__Adlercreutzia;s__	p__Actinobacteria;c__Coriobacteriia; o__Coriobacteriales;f__Coriobacteriaceae; g__;s__
	p__Bacteroidetes;c__Bacteroidia; o__Bacteroidales;f__Bacteroidaceae; g__Bacteroides;__	p__Bacteroidetes;c__Bacteroidia; o__Bacteroidales;f__Bacteroidaceae; g__Bacteroides;s__ovatus
	p__Bacteroidetes;c__Bacteroidia; o__Bacteroidales;f__Bacteroidaceae; g__Bacteroides;s__	p__Bacteroidetes;c__Bacteroidia; o__Bacteroidales;f__Bacteroidaceae; g__Bacteroides;s__uniformis
	p__Bacteroidetes;c__Bacteroidia; o__Bacteroidales;f__Bacteroidaceae; g__Bacteroides;s__acidifaciens	p__Deferribacteres;c__Deferribacteres; o__Deferribacterales;f__Deferribacteraceae; g__Mucispirillum;s__schaedleri
	p__Bacteroidetes;c__Bacteroidia; o__Bacteroidales;f__Porphyromonadaceae; g__Parabacteroides;s__	p__Firmicutes;c__Bacilli; o__Lactobacillales;f__Enterococcaceae; g__Enterococcus;__
	p__Bacteroidetes;c__Bacteroidia; o__Bacteroidales;f__Porphyromonadaceae; g__Parabacteroides;s__distasonis	p__Firmicutes;c__Clostridia; o__Clostridiales;f__Lachnospiraceae; g__Dorea;s__
	p__Bacteroidetes;c__Bacteroidia; o__Bacteroidales;f__Porphyromonadaceae; g__Parabacteroides;s__gordonii	p__Firmicutes;c__Erysipelotrichi; o__Erysipelotrichales;f__Erysipelotrichaceae; g__Coprobacillus;s__
	p__Bacteroidetes;c__Bacteroidia; o__Bacteroidales;f__Prevotellaceae; g__Prevotella;s__	p__Proteobacteria;c__Deltaproteobacteria; o__Desulfovibrionales;f__Desulfovibrionaceae; g__;s__
	p__Bacteroidetes;c__Bacteroidia; o__Bacteroidales;f__S24-7; g__;s__	p__Proteobacteria;c__Epsilonproteobacteria; o__Campylobacterales;f__Helicobacteraceae;__;__
	p__Bacteroidetes;c__Bacteroidia; o__Bacteroidales;f__[Paraprevotellaceae]; g__[Prevotella];s__	p__Verrucomicrobia;c__Verrucomicrobiae; o__Verrucomicrobiales;f__Verrucomicrobiaceae; g__Akkermansia;s__muciniphila
	p__Firmicutes;c__Bacilli; o__Lactobacillales;f__Lactobacillaceae; g__Lactobacillus;__	
	p__Firmicutes;c__Bacilli; o__Lactobacillales;f__Lactobacillaceae; g__Lactobacillus;s__	
	p__Firmicutes;c__Bacilli; o__Lactobacillales;f__Streptococcaceae; g__Streptococcus;s__	
	p__Firmicutes;c__Bacilli; o__Lactobacillales;f__Streptococcaceae; g__Streptococcus;s__luteciae	

Primers sequences associated with the seven PCR amplicons covering 9 variable regions in the bacterial 16S rRNA gene are listed below.

**Table:** 

Name	Sequence
V1-V2_F	TCGTCGGCAGCGTCAGAGTTTGATCCTGGCTCAG
V2_F	TCGTCGGCAGCGTCAGYGGCGIACGGGTGAGTAA
V3_2_F	TCGTCGGCAGCGTCCCTACGGGAGGCAGCAG
V4_F	TCGTCGGCAGCGTCGTGCCAGCMGCCGCGGTAA
V5-V6_F	TCGTCGGCAGCGTCAGGATTAGATACCCTGGTA
V6_1_F	TCGTCGGCAGCGTCAAACTCAAAKGAATTGACGG
V7-V8_F	TCGTCGGCAGCGTCGYAACGAGCGCAACCC
V1-V2_R	GTCTCGTGGGCTCGGTGCTGCCTCCCGTAGGAGT
V2_R	GTCTCGTGGGCTCGGCYIACTGCTGCCTCCCGTAG
V3_2_R	GTCTCGTGGGCTCGGGTATTACCGCGGCTGCTGG
V4_R	GTCTCGTGGGCTCGGGGACTACHVGGGTWTCTAAT
V5-V6_R	GTCTCGTGGGCTCGGCRRCACGAGCTGACGAC
V6_1_R	GTCTCGTGGGCTCGGACGAGCTGACGACARCCATG
V7-V8_R	GTCTCGTGGGCTCGGGACGGGCGGTGWGTRC

### WashU and U. Chicago Luminex cytokine/chemokine array

Mouse serum and brain tissue lysate samples were thawed on ice, centrifuged at 15,000 rcf for 10 min at 4C to remove particulates and aggregates, then 25 uL of sample or prepared kit standard was added to each well (in duplicate) of a 96 well plate containing premixed beads and assay buffer. The standard for the tissue lysates was prepared with the lysis buffer (Invitrogen ProcartaPlex cell lysis buffer). The bead-based multiplex immunoassay was performed according to the manufacturer’s instructions (ThermoFisher Procartaplex Mouse Cytokine/Chemokine Panel 1A 36plex; # EPXR360-26092–901). The panel probed for the following chemokines and cytokines: ENA-78 (CXCL5), Eotaxin (CCL11), GRO alpha (CXCL1), IP-10 (CXCL10), MCP-1 (CCL2), MIP-1 alpha (CCL3), MIP-1 beta (CCL4), MIP-2 alpha (CXCL2), RANTES (CCL5) Cytokines: G-CSF (CSF-3), GM-CSF, IFN alpha, IFN gamma, IL-1 alpha, IL-1 beta, IL-2, IL-3, IL-4, IL-5, IL-6, IL-9, IL-10, IL-12p70, IL-13, IL-15/IL-15R, IL-17A (CTLA-8), IL-18, IL-22, IL-23, IL-27, IL-28, IL-31, LIF, MCP-3 (CCL7), M-CSF, TNF alpha. The plate was incubated on a shaker at 700 rpm for 2 h at room temperature, washed on a hand-held magnet, then the detection antibody was added for 30 min. After washing the unbound detection antibody away from the beads, the streptavidin phycoerythrin (SA-PE) reagent was added to each well and the plate shaken for 30 min. A final wash was performed then the beads were read for MFI using a FLEXMAP3D Luminex (Luminex Corp, Austin, TX) machine. MilliporeSigma Belysa v.1 (Merck EMD Millipore, Billerica, MA) analysis software was used to calculate the pg/ml for each analyte using a 5-parameter logistical curve-fit algorithm.

### Metabolite extraction from cecal material

Extraction solvent (80% methanol spiked with internal standards and stored at -80 °C) was added to pre-weighed fecal/cecal samples at a ratio of 100 mg of material/mL of extraction solvent in beadruptor tubes (Fisherbrand; 15–340-154). Samples were homogenized at 4 °C on a Bead Mill 24 Homogenizer (Fisher; 15–340-163), set at 1.6 m/s with 6 thirty-second cycles, 5 s off per cycle. Samples were then centrifuged at -10 °C, 20,000 × g for 15 min and the supernatant was used for subsequent metabolomic analysis*.*

### Metabolite analysis using GC-nCI-MS and PFBBr derivatization

Metabolites were derivatized as described by Haak et al. [26] with the following modifications: the metabolite extract (100μL) was added to 100μL of 100 mM borate buffer (pH 10) (Thermo Fisher, #28341), 400μL of 100 mM pentafluorobenzyl bromide (Millipore Sigma; #90257) in acetonitrile (Fisher; A955-4), and 400μL of n-hexane (Acros Organics; #160780010) in a capped mass spec autosampler vial (Microliter; 09–1200). Samples were heated in a Thermomixer C (Eppendorf) to 65 °C for 1 h while shaking at 1300 rpm. After cooling to RT, samples were centrifuged at 4 °C, 2000 × g for 5 min, allowing phase separation. The hexane phase (100μL) (top layer) was transferred to an autosampler vial containing a glass insert and the vial was sealed. Another 100μL of the hexane phase was diluted with 900μL of n-hexane in an autosampler vial. Concentrated and dilute samples were analyzed using a GC–MS (Agilent 7890A GC system, Agilent 5975C MS detector) operating in negative chemical ionization mode, using a HP-5MSUI column (30 m × 0.25 mm, 0.25 μm; Agilent Technologies; #19091S-433UI), methane as the reagent gas (99.999% pure) and 1μL split injection (1:10 split ratio). Oven ramp parameters: 1 min hold at 60 oC, 25 oC per min up to 300 °C with a 2.5 min hold at 300 °C. Inlet temperature was 280 °C and transfer line was 310 °C. A 10-point calibration curve was prepared with acetate (100 mM), propionate (25 mM), butyrate (12.5 mM), and succinate (50 mM), with 9 subsequent 2x serial dilutions. Data analysis was performed using MassHunter Quantitative Analysis software (version B.10, Agilent Technologies) and confirmed by comparison to authentic standards. Normalized peak areas were calculated by dividing raw peak areas of targeted analytes by averaged raw peak areas of internal standards.

### LCMS/MS

Indole-containing metabolites, B-vitamins and other targeted metabolites were analyzed by LCMS/MS. The metabolite extract (400μL) was added to pre-labeled microcentrifuge tubes. Samples were dried down completely using a Genevac EZ-2 Elite. Samples were resuspended in 100μL of 50:50 Water: Methanol and added to an Eppendorf thermomixer. C at 4 oC, 1000 rpm for 15 min to resuspend analytes. Samples were then centrifuged at 4 °C, 20,000 × g for 15 min to remove insoluble debris. The supernatant (80μL) was transferred to a fresh, prelabeled MS vial with inserts or 96 deep-well plate (Agilent; #5065–4402). Samples were analyzed on an Agilent 1290 infinity II liquid chromatography system coupled to an Agilent 6470 triple quadrupole mass spectrometer, operating in positive mode, equipped with an Agilent Jet Stream Electrospray Ionization source. Each sample (2μL) was injected into a Acquity UPLC HSS PFP column, 1.8 μm, 2.1 × 100 mm (Waters; 186005967) equipped with a Acquity UPLC HSS PFP VanGuard Precolumn, 100., 1.8 μm, 2.1 mm X 5 mm (Waters; 186005974) at 45 oC. Mobile phase A was 0.35% formic acid in Water and mobile phase B was 0.35% formic acid in 95:5 Acetonitrile:Water. The flow rate was set to 0.5 mL/min starting at 0% B held constant for 3 min, then linearly increased to 50% over 5 min, then linearly increased to 95% B over 1 min, and held at 100% B for the next 3 min. Mobile phase B was then brought back down to 0% over 0.5 min and held at 0% for re equilibration for 2.5 min. The QQQ electrospray conditions were set with capillary voltage at 4 kV, nozzle voltage at 500 V, and Dynamic MRM was used with cycle time of 500 ms. Transitions were monitored in positive mode for 46 analytes (table on next slide). An 11-point calibration curve (ranging from 0.88 nM to 909 μM) was prepared for tryptophan, tyrosine, phenylalanine, serotonin, 5-HIAA, melatonin, tryptamine, kynurenine, kynurenic acid, anthranilic acid, and niacin. Data analysis was performed using MassHunter Quant software (version B.10, Agilent Technologies) and confirmed by comparison with authentic standards. Normalized peak areas were calculated by dividing raw peak areas of targeted analytes by averaged raw peak areas of internal standards.

### Bile acid analysis

Bile acids were analyzed using LCMS. The metabolite extract (75μL) was added to prelabeled mass spectrometry autosampler vials (Microliter; 09–1200) and dried down completely under a nitrogen stream at 30 L/min (top) 1 L/min (bottom) at 30 °C (Biotage SPE Dry 96 Dual; #3579 M). Samples were resuspended in 50:50 Water: Methanol (750μL). Vials were added to a thermomixer C (Eppendorf) to resuspend analytes at 4 °C, 1000 rpm for 15 min with an infinite hold at 4 °C. Samples were then transferred to prelabeled microcentrifuge tubes and centrifuged at 4 °C, 20,000 × g for 15 min to remove insoluble debris. The supernatant (700μL) was transferred to a fresh, prelabeled mass spectrometry autosampler vial. Samples were analyzed on a liquid chromatography system (Agilent 1290 infinity II) coupled to a quadrupole time-of-flight (QTOF) mass spectrometer (Agilent 6546), operating in negative mode, equipped with an Agilent Jet Stream Electrospray Ionization source. The sample (5μL) was injected onto an XBridge© BEH C18 Column (3.5 μm, 2.1 × 100 mm; Waters Corporation, PN) fitted with an XBridge© BEH C18 guard (Waters Corporation, PN) at 45 °C. Elution started with 72% A (Water, 0.1% formic acid) and 28% B (Acetone, 0.1% formic acid) with a flow rate of 0.4 mL/min for 1 min and linearly increased to 33% B over 5 min, then linearly increased to 65% B over 14 min. Then the flow rate was increased to 0.6 mL/min and B was increased to 98% over 0.5 min and these conditions were held constant for 3.5 min. Finally, re-equilibration at a flow rate of 0.4 mL/min of 28% B was performed for 3 min. The electrospray ionization conditions were set with the capillary voltage at 3.5 kV, nozzle voltage at 2 kV, and detection window set to 100–1700 m/z with continuous infusion of a reference mass (Agilent ESI TOF Biopolymer Analysis Reference Mix) for mass calibration. A ten-point calibration curve was used for quantitation. Data analysis was performed using MassHunter Profinder Analysis software (version B.10, Agilent Technologies) and confirmed by comparison with authentic standards. Normalized peak areas were calculated by dividing raw peak areas of targeted analytes by averaged raw peak areas of internal standards.

### Statistical analysis

We used GraphPad Prism (version 7.0e) to run statistical analyses. Two-way ANOVA were performed to evaluate different parameters among vehicle vs treatment groups in a sex-specific manner unless otherwise noted. For the 5XFAD study, sexes were separated and student t-test was utilized to compare GV-971 with controls. Statistical *P* value below 0.05 was considered for significant differences unless otherwise noted. For microbiome data analysis, Kruskal–Wallis (non-parametric) comparisons were performed to compare several diversity-related indices. ANCOM was performed as mentioned above to evaluate the differences at species level between groups. QIIME and QIIME2 was utilized to determine diversity analyses, including alpha and β diversity, alpha rarefaction, and group significance for the 5XFAD microbiome analysis.

## Results

### GV-971 treatment results in a sex-dependent reduction of Aβ amyloidosis in amyloid-depositing mice from two independent institutions

Aβ plaque deposition is a pathological hallmark of AD and is associated with the onset of neuroinflammation. Recent publications from our labs have shown a direct correlation between the gut microbiome and the severity of amyloid beta plaque deposition and phosphorylated tau accumulation [19, 46]. To investigate the effects of GV-971 on cerebral amyloidosis, researchers at the University of Chicago (U.Chicago) and Washington University (WashU) in St. Louis conducted two independent experimental paradigms unaware of the experiments that each group was doing until after the main data collection had been completed. It is important to note that the microbiome composition from the same strain of amyloid-depositing mice significantly differs between the universities (Figure S1). The alpha and beta diversities indicate separate bacterial populations (Figure S1a, b, c, d). Therefore, to determine if GV-971 can alter cerebral amyloidosis in mice with differing microbiomes, U. Chicago investigated APPPS1-21 mice, while WashU investigated 5XFAD mice. Both are validated models of cerebral amyloidosis and neuroinflammation.

**Fig. 1:**
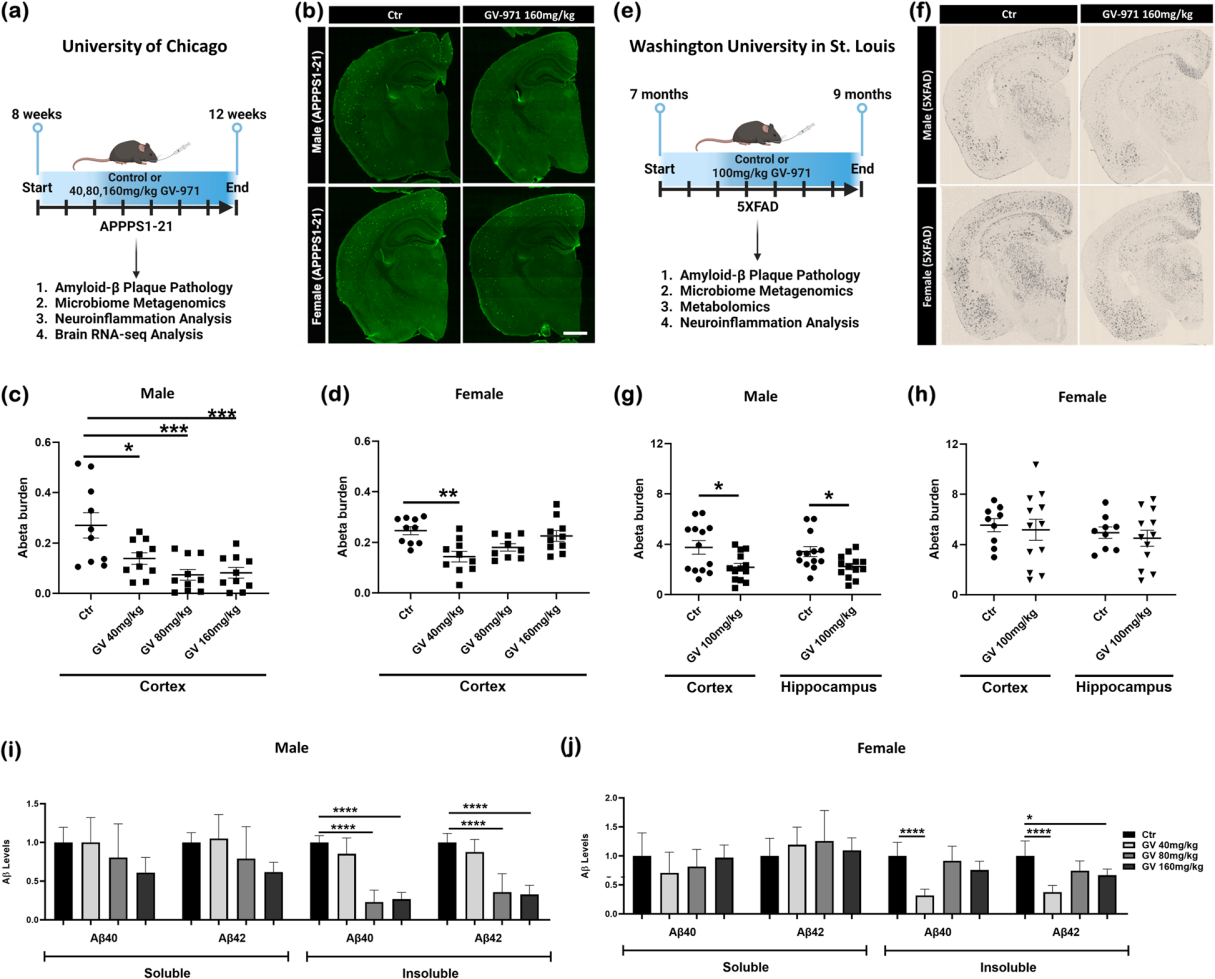
GV-971 reduces amyloidosis in a dose and sex-dependent manner. **a** University of Chicago experimental design. **b** Representative images of brains stained with anti-Aβ antibody 3D6 depicting the effect of GV-971 on Aβ plaque burden in APPPS1-21 mice. **c** Quantification of % area covered by 3D6^+^ Aβ plaques in cortices of APPPS1-21 male mice (*n* = 10). **d** Quantification of % area covered by 3D6^+^ Aβ plaques in cortices of APPPS1-21 female mice (*n* = 9–11). **e** Washington University in St. Louis experimental design. **f** Representative images of brains stained with anti-Aβ antibody HJ3.4 showing the effect of GV-971 on Aβ plaque burden in 5XFAD mice. **g** Quantification of % area covered by HJ3.4^+^ Aβ plaques in cortices of 5XFAD male mice (*n* = 13). **h** Quantification of % area covered by HJ3.4^+^ Aβ plaques in cortices of 5XFAD female male (*n* = 9–12). **i** Quantification of insoluble and soluble Aβ 40 and 42 isoforms extracted from cortical tissue of male APPPS1-21 mice (*n* = 10). **j** Quantification of insoluble and soluble Aβ 40 and 42 isoforms extracted from cortical tissue of female APPPS1-21 mice (*n* = 9–11). Data presented as SEM. Significance determined using a One-way ANOVA test followed by Tukey’s multiple comparison post hoc test (**c**, **d**, **i**, **j**), and unpaired t-test (**g**, **h**). *, *P* < 0.05; **, *P* < 0.01; ***, *P* < 0.001; ****, *P* < 0.0001

In APPPS1-21 mice, we orally administered 40, 80, and 160 mg/kg of GV-971 to 2-month-old male and female APPPS1-21 mice for one month (Fig. 1a). The brains were collected and cortical Aβ burden was assessed using threshold-based analysis. Interestingly, we found that GV-971 treatment resulted in a significantly lower cortical Aβ burden in male mice (Fig. 1b, c), while female mice showed a significant reduction in Aβ burden at the lower dose; however, this reduction was not sustained at the higher doses (Fig. 1b, d). Moreover, the male 80 mg/kg GV-971-treated male group showed the most prominent reduction in Aβ burden, with no further decrease of Aβ burden at 160 mg/kg. However, there was no significant difference in Aβ burden in the brains of animals when comparing 40, 80, and 160 mg/kg of GV-971.

Previous clinical studies examining the efficacy of GV-971 found significant cognitive improvement in Alzheimer’s disease patients with mild to moderate cognitive impairment, a time when patients have heavy Aβ deposition within the brain. Therefore, we sought to assess the effects of GV-971 by utilizing the 5XFAD amyloidosis model at 7 months of age, when mice have significant Aβ plaque deposition and severe neuroinflammation. We orally administered 7-month-old male and female 5XFAD mice with 100 mg/kg GV-971, a dose comparable to that used in previous studies and clinical trials [17]. The animals were collected at 2 months post-treatment, and cortical Aβ burden was assessed using threshold-based analysis (Fig. 1e). Similar to what was found in the APPPS1-21 mice, GV-971 significantly reduced cortical and hippocampal Aβ plaque burden in male mice, while female mice displayed no significant changes (Fig. 1f, g, h). To further investigate the effect GV-971 is having on Aβ plaque pathology at later time points, we characterized plaque morphology utilizing two methods: a stain for amyloid fibrils that target dense fibrillary plaque cores, and a pan anti-Aβ antibody that targets amino acids 1–6 of Aβ. Male mice treated with 100 mg/kg GV-971 had significantly less pan amyloid staining within 5 μm of the fibrillary plaque core (Figure S2a, b). There were no differences in plaque morphology in treated female mice (Figure S2a, c). These data indicate that GV-971 specifically affected the Aβ plaque halo in the presence of substantial plaque deposition.

To further characterize changes in amyloid protein species, we then extracted soluble and insoluble Aβ1-40 and Aβ1-42 from APPPS1-21 ventral cerebral cortices and measured levels of Aβ isoforms by the meso scale discovery (MSD) ELISA platform. We observed no significant differences in soluble Aβ levels in GV-treated mice at any dosage tested, regardless of sex. However, insoluble Aβ1-40 and Aβ1-42 levels were significantly reduced in male GV 80 mg/kg and 160 mg/kg treatment groups (Fig. 1i, j). In extracts prepared from the brains of female mice, we observed a significant reduction in insoluble Aβ1-40 and Aβ1-42 levels in the 40 mg/kg treatment group, but there was no impact of GV-971 on insoluble Aβ levels in the 80 and 160 mg/kg treatment group compared with vehicle-treated controls (Fig. 1i, j). Therefore, GV-971 treatment reduces Aβ amyloidosis in APPPS1-21 mice in a sex-specific manner.

### GV-971 treatment results in gut microbiome changes in male and female mice

**Fig. 2:**
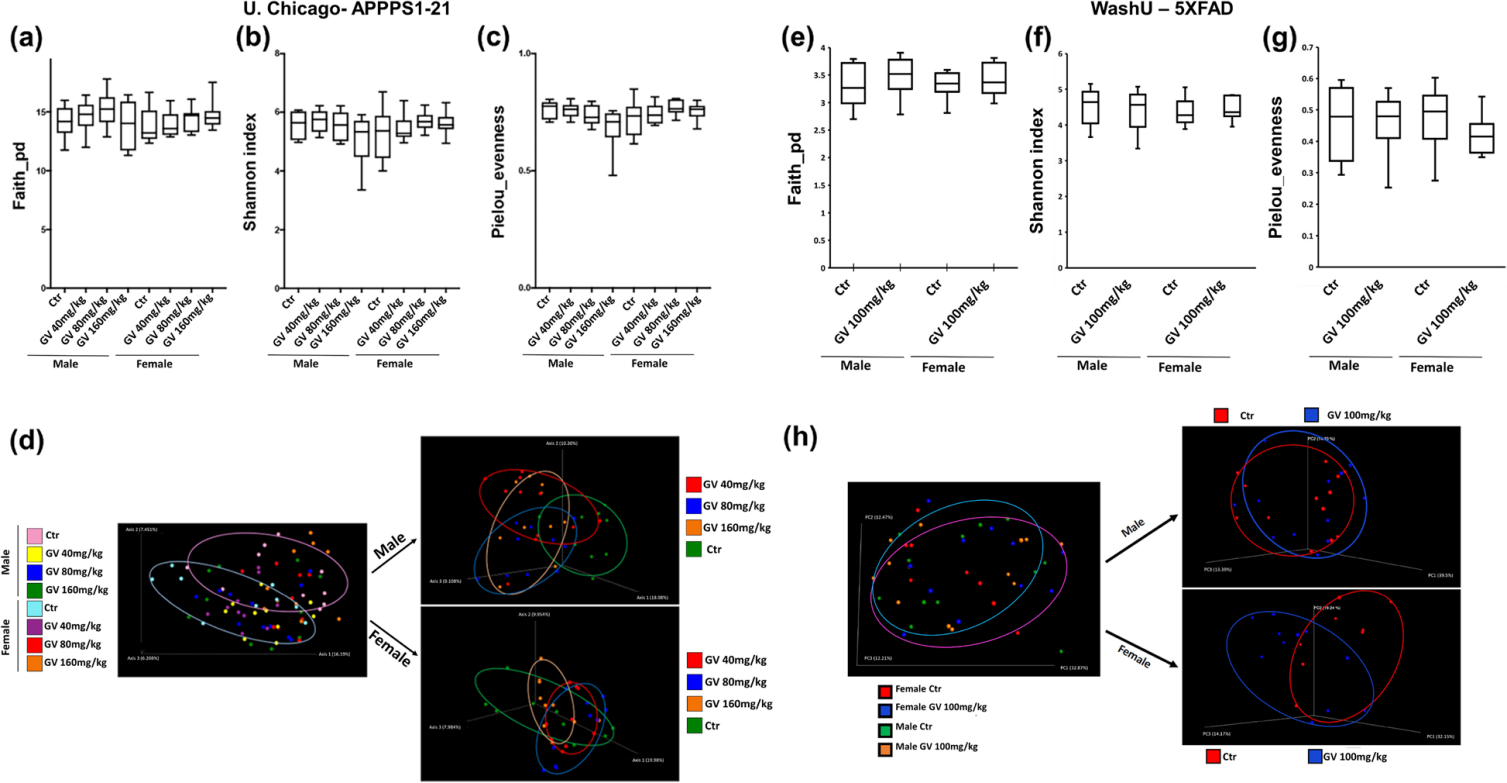
GV-971 alters microbiome β-diversity in APPPS1-21 male mice. Analysis of bacterial α-diversity from cecal content of University of Chicago APPPS1-21 mice. **a** Faith phylogenetic diversity, **b** Shannon index, **c** Pielou species evenness. **d** PCoA plot generated by using unweighted unifrac distance metric. Analysis of bacterial α-diversity from cecal content of Washington University in St. Louis 5XFAD mice. **e** Faith phylogenetic diversity, **f** Shannon index, **g** Pielou species evenness. **h** PCoA plot generated by using unweighted unifrac distance metric. Diversity analyses, including alpha and beta diversity, alpha rarefaction, and group significance were analyzed by QIIME and QIIME2

It was previously found that GV-971 alters gut microbiome composition that was correlated with a reduction in multiple inflammatory pathways. Cecal content and fecal specimens were collected at the time of sacrifice to investigate the impact of GV-971 treatment on microbiota changes. To understand if GV-971 influences microbiota in a sex-specific manner, we separated male and female groups (Fig. 2a-h). Analysis of microbial alpha-diversity revealed no significant changes among phylogenetic diversity indices Faith (Fig. 2a), Shannon diversity (Fig. 2b), or evenness (Fig. 2c) among all groups of APPPS1-21 mice. Compared with vehicle-treated male groups, female groups showed significant differences, as seen by separated clusters in Fig. 2d (PERMANOVA, *P* = 0.013), indicating sex-specific differences in microbiome composition. Moreover, within male groups, analysis of β-diversity using the Unweighted UniFrac metric showed significant differences among all groups compared with the vehicle-treated male group (PERMANOVA: Male_Ctr vs Male_GV40 mg/kg *P* = 0.002; Male_Ctr vs Male_GV80mg/kg *P* = 0.012; Male_Ctr vs Male_GV160 mg/kg *P* = 0.028). In contrast, female groups only showed significant differences between vehicle-treated females compared with GV80 mg/kg and GV160 mg/kg (PERMANOVA: Female_Ctr vs Female_GV80 mg/kg *P* = 0.018; Female_Ctr vs Female_GV80 mg/kg *P* = 0.001). It is important to note that the significant changes in β-diversity from GV-971 treatment shift microbiota independent of sex.

We then investigated the effects of GV-971 on 9-month-old 5XFAD mice by performing 16 s rRNA amplicon sequencing on clean catch fecal samples. Similar to APPPS1-21 mice, analysis of microbial alpha-diversity revealed no significant changes among phylogenetic diversity indices Faith (Fig. 2e), Shannon diversity (Fig. 2f), or evenness (Fig. 2g) among all groups. Additionally, there was no significant shift in the β-diversity using the Unweighted uniFrac measurements in either sex (Fig. 2h). In summary, GV-971 significantly changed the β-diversity in only APPPS1-21 mice.

Interestingly, we observed significant alterations in the abundance of microbial species in both APPPS1-21 and 5XFAD mice treated with GV-971. To further investigate these changes in both mouse models, we conducted an analysis of the composition of microbes (ANCOM for APPPS1-21 mice and MVISION and QIIME for 5XFAD mice) to identify amplicon sequence variants (ASVs) with significantly different proportions in the APPPS1-21 160 mg/kg treatment group as well as the 5XFAD 100 mg/kg treated group [34, 47]. The 160 mg/kg treatment group was selected for future analysis as it had a profound change in the microbiota profiles compared to control. Firstly, we observed significant changes in cecal microbiota profiles in maleAPPPS1-21 mice treated with GV-971 160 mg/kg compared with the control male group (Table 1).

Among them, the GV 160 mg/kg treated group exhibited a larger proportion of increased ASVs such as: in the order Bifidobacterials (phylum Actinobacteria) pseudolongum sp., in the order Bacteroidales (phylum Bacteroidetes), acidifaciens sp., in the order clostridiales (phylum Firmicutes), obeum sp, in the order Desulfovibrionaceae (phylum Proteobacteria) and several other as listed in Table 1. Bacteria exhibiting a decrease in GV 160 mg/kg-treated group relative to vehicle-treated males included Bacteroides Uniformis (Phylum Bacteroidetes), genus Corynebacterium and family Coriobacteriaceae (phylum Actinobacteria), Enterococcus (phylum Firmicutes) and few others as listed in Table 1. In contrast, female groups showed very few significantly altered taxa following treatment with 160 mg/kg GV-971 (Table 2). The GV 160 mg/kg female group showed a reduced abundance of Ruminococcus flavefaciens sp. (phylum Firmicutes) and peptostreptococcaceae family (phylum Firmicutes).

**Table 2:** GV-971 significantly alters microbiome bacterial species in female mice treated with 160 mg/kg compared with vehicle

**Comparisons**		
**Female Ctrl vs Female 160 mg/kg GV-971**	**Increased in GV-971**	**Decreased in GV-971**
	p__Firmicutes;c__Clostridia; o__Clostridiales; f__Peptostreptococcaceae; g__;s__	There were no signficant decreased bacteria
	p__Firmicutes;c__Clostridia; o__Clostridiales; f__Ruminococcaceae; g__Ruminococcus;s__flavefaciens	

While no significant diversity changes were found in the 5XFAD mice microbiome comparing GV-971 100 mg/kg to vehicle (Fig. 2h), there were changes in bacterial species abundance. Male mice treated with GV-971 presented with a greater abundance of ASVs in the order Bifidobacteriales (phylum Actinobacteria), Bifidobacterium pseudolongum sp., in the order Erysipelotrichales (phylum Firmicutes) Faecalibaculum rodentium sp., in the order Burkholderiales (phylum Protobacteria) Parasutterella excrementihominis sp. Bacteria exhibiting a decrease in abundance in GV-971 treated male treated mice include in the order Clostridiales (phylum Firmicutes) G32012 sp., in the order Desulfovibrionaceae (phylum Proteobacteria) and in the order of Rikenellaceae (phylum Bacteroidetes) Alistipes onderdonkii sp (Table 3). Similar to the APPPS1-21 female mice, there were few taxa changes in the female 5XFAD GV-971 treated mice compared to the vehicle. These included increases in the order of Bacteroidales (phylum Bacteroidetes) YL27 sp, in the order of Eggerthellales (phylum Actinobacteria) Entevacteroides mucosicola sp, and a decrease in the order of Clostridales (phylum Firmicutes) Rumivacteroides leptum sp (Table 4). Collectively, these data indicate that GV-971 treatment resulted in sex-specific microbiota differences with the most prominent changes in the gut microbiota of male APPPS1-21 mice treated at early time points or 5X FAD mice treated at late time points.

**Table 3:** GV-971 significantly alters microbiome bacterial species in male mice treated with 100 mg/kg compared with vehicle

**Comparisons**		
**Male Ctrl vs Male 100 mg/kg GV- 971**	**Increased in GV-971**	**Decreased in GV-971**
	p__Actinobacteria;c__Actinobacteria; o__Bifidobacteriales;f__Bifidobacteriaceae; g__Bifidobacterium;s__Bifidobacterium pseudolongum	p__Firmicutes;c__Clostridia; o__Clostridiales;f__Ruminococcaceae; g__Anaerotruncus;s__Anaerotruncus sp G32012
	p__Firmicutes;c__Erysipelotrichia; o__Erysipelotrichales;f__Erysipelotrichaceae; g__Faecalibaculum;s__Faecalibaculum rodentium	p__Proteobacteria;c__Deltaproteobacteria; o__Desulfovibrionales;f__Desulfovibrionaceae; g__Desulfovibrio;s__Desulfovibrio sp ABHU2SB
	p__Proteobacteria;c__Betaproteobacteria; o__Burkholderiales;f__Sutterellaceae; g__Parasutterella;s__Parasutterella excrementihominis	p__Bacteroidetes;c__Bacteroidia; o__Bacteroidales;f__Rikenellaceae; g__Alistipes;s__Alistipes onderdonkii
		p__Firmicutes;c__Clostridia; o__Clostridiales;f__Lachnospiraceae; g__Blautia;s__Ruminococcus gnavus
		p__Firmicutes;c__Clostridia; o__Clostridiales;f__Ruminococcaceae; g__Ruminiclostridium;s__Clostridium leptum

**Table 4:** GV-971 significantly alters microbiome bacterial species in female mice treated with 100 mg/kg compared with vehicle

**Comparisons**		
**Female Ctrl vs Female 100 mg/kg GV-971**	**Increased in GV-971**	**Decreased in GV-971**
	p__Bacteroidetes;c__Bacteroidia; o__Bacteroidales;f__Tannerellaceae; g__Parabacteroides;s__Parabacteroides sp YL27	p__Firmicutes;c__Clostridia; o__Clostridiales;f__Clostridiaceae; g__Clostridium;s__Clostridium sp Clone16
	p__Firmicutes;c__Clostridia; o__Clostridiales;f__Ruminococcaceae; g__Ruminiclostridium;s__Clostridium leptum	p__Actinobacteria;c__Coriobacteriia; o__Eggerthellales;f__Eggerthellaceae; g__Enterorhabdus;s__Enterorhabdus mucosicola
		p__Firmicutes;c__Clostridia; o__Clostridiales;f__Lachnospiraceae; g__Blautia;s__Blautia coccoides
		p__Firmicutes;c__Clostridia; o__Clostridiales;f__Clostridiaceae; g__Clostridium;s__Clostridium sp Clone49
		p__Firmicutes;c__Clostridia; o__Clostridiales;f__Clostridiaceae; g__Clostridium;s__Clostridium sp ASF502
		p__Firmicutes;c__Clostridia; o__Clostridiales;f__Clostridiaceae; g__Clostridium;s__Clostridium sp MarseilleP2776

### GV-971 alters microbiome metabolism resulting in changes in amino acids, tryptophan and bile acid production

**Fig. 3:**
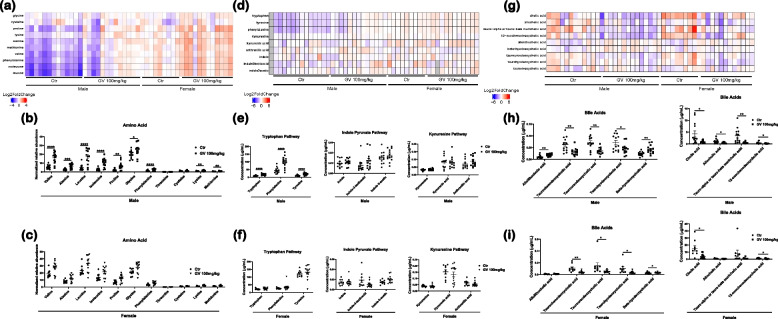
GV-971 alters microbiome metabolism in 5XFAD mice. **a** Representative heat map of significant amino acids abundance in cecal content of 5XFAD mice treated with 100 mg/kg GV-971 or vehicle from Washington University in St. Louis. **b** Quantification of amino acid abundance in cecal content of 5XFAD male mice (*n* = 13). **c** Quantification of amino acid abundance in cecal content of 5XFAD female mice (*n* = 9–12). **d** Representative heat map of significant metabolites in the tryptophan, indole pyruvate, and kynurenine pathway. **e** Quantification of tryptophan, indole pyruvate, and kynurenine pathway metabolite concentrations in cecal content of 5XFAD male mice (*n* = 13). **f** Quantification of tryptophan, indole pyruvate, and kynurenine pathway metabolite concentrations in cecal content of 5XFAD female mice (*n* = 9–12). **g** Representative heat map of significant primary and secondary bile acids. **h** Quantification of primary and secondary bile acids concentrations in cecal content of 5XFAD male mice (*n* = 13). **i** Quantification of primary and secondary bile acids concentrations in cecal content of 5XFAD female mice (*n* = 9–12). Data presented as SEM. Significance determined using unpaired t-test. *, *P* < 0.05; **, *P* < 0.01; ***, *P* < 0.001; ****, *P* < 0.0001

The gut microbiome plays a crucial role in regulating metabolic functions, such as digestion and absorption of metabolites, while also producing key metabolites such as short-chain fatty acids [48]. These short-chain fatty acids not only regulate immune functions but also facilitate communication along the gut-brain axis [7]. As GV-971 altered the gut microbiota diversity and specific species abundance, we chose to conduct metabolomic analysis on cecal content from 5XFAD-treated mice. First, we performed pentafluorobenzyl bromide gas chromatography–mass spectrometry to identify changes in multiple metabolic pathways including short-chain fatty acid, amino acid, and fatty acid metabolism. There were no significant changes in short-chain fatty acid levels in both sexes treated with GV-971 (Figure S3). However, multiple vital amino acids were significantly increased in 5XFAD male mice following treatment with GV-971 (Fig. 3a, b). These include valine, alanine, leucine, isoleucine, proline, glycine, phenylalanine, lysine, and methionine (Fig. 3a, b). There were no changes in amino acid abundance in 5XFAD female mice (Fig. 3a, c). Previous studies have shown the critical role of tryptophan metabolism for both CNS and immune functions. Tryptophan serves a precursor to the neurotransmitter serotonin, playing a role in neurotransmission and enteric functions [49]. Next, we performed targeted LCMS/MS to identify changes in tryptophan metabolism. GV-971 significantly increased tryptophan metabolism in only 5XFAD male mice compared to the control (Fig. 3d, e). There were no significant changes observed in any of these pathways in female mice treated with GV-971 (Fig. 3d, f). Despite observing increased tryptophan levels in male mice, there were no changes in downstream pathways such as indole or kynurenine metabolism (Fig. 3d, e, f). Finally, it is known that the gut microbiome has a bidirectional relationship with bile acid production. Changes in bile acid composition have been connected with dysbiosis [50]. Primary and secondary bile acids were analyzed using LCMS/MS. GV-971 significantly increased Allolithocholic acid and Beta-hyodeoxycholic acid, while simultaneously significantly reduced Taurochenodeoxycholic acid, Tauroursodeoxycholic acid, Taurohyodeoxycholic acid,, Cholic acid, Allocholic acid, Tauro-alpha or tauro-beta muricholic acid, and 12-oxochenodeoxycholic acid in male 5XFAD mice compared with vehicle-treated controls (Fig. 3g, h). GV-971 significantly reduced Taurochenodeoxycholic acid, Tauroursodeoxycholic acid, Taurohyodeoxycholic acid, Beta-hyodeoxycholic acid, Cholic acid, and 12-oxochenodeoxycholic acid in female 5XFAD mice compared with vehicle-treated controls (Fig. 3g, i). For full MS panel analysis refer to Figures S3, S4 and S5. In summary, this data demonstrated that GV-971 affects the gut microbiome and subsequent metabolic profiles in 5XFAD mice. Specifically, GV-971 leads to significant alterations in amino acid and tryptophan metabolism in 5XFAD male mice, while also impacting bile acid composition.

### GV-971 modulates cytokine and chemokine levels in both the periphery and cortical tissue

**Fig. 4:**
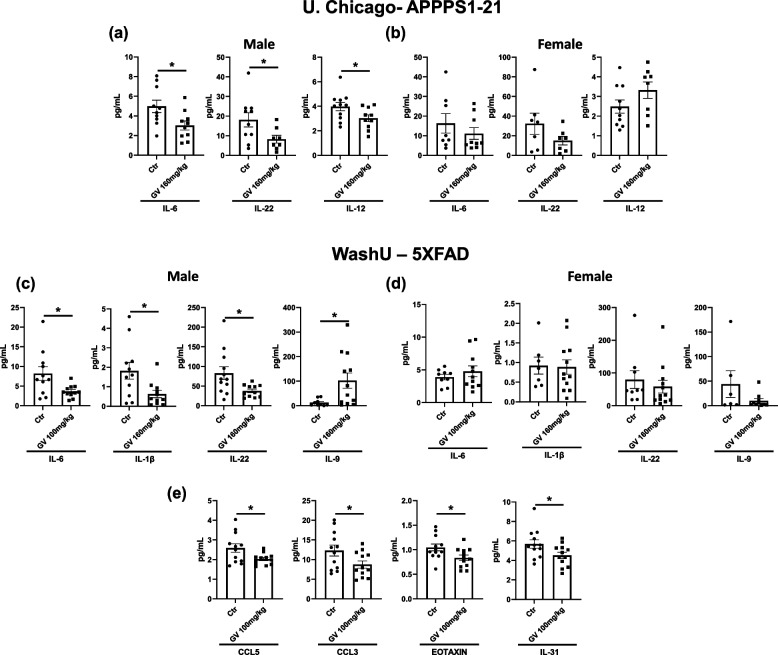
GV-971 modifies cytokine and chemokine levels in peripheral blood and cortical tissues. **a** Quantification of cytokine and chemokine concentrations in the serum of APPPS1-21 male mice treated with 160 mg/kg GV-971 or vehicle from the University of Chicago (*n* = 10–11). **b** Quantification of cytokine and chemokine concentrations in the serum of APPPS1-21 female mice treated with 160 mg/kg GV-971 or vehicle (*n* = 8–10). **c**, **d** Quantification of cytokine and chemokine concentrations in the serum of 5XFAD male mice treated with 100 mg/kg GV-971 or vehicle from Washington University in St. Louis (*n* = 12–13). **e**, **f** Quantification of cytokine and chemokine concentrations in the serum of 5XFAD female mice treated with 100 mg/kg GV-971 or vehicle (*n* = 9–12). **g** Quantification of cytokine and chemokine concentrations in the cortical tissue of 5XFAD male mice treated with 100 mg/kg GV-971 or vehicle (*n* = 12–13). Data presented as SEM. Significance determined using unpaired t-test. *, *P* < 0.05; **, *P* < 0.01; ***, *P* < 0.001; ****, *P* < 0.0001

The gut microbiome has a close relationship with the peripheral immune system, with the ability to signal immune cells and regulate both innate and adaptive immune responses [51]. Gut dysbiosis has been implicated in multiple autoimmune diseases and is a primary driver of local inflammation [52]. Since we established that GV-971 altered a significant number of commensal bacterial species (Tables 1, 2, 3 and 4), we next quantified the levels of cytokines and chemokines circulating in the blood and cortical brain tissue. Serum was collected from male and female APPPS1-21 and 5XFAD mice, and peripheral cytokine/ chemokine analytes were measured using a Luminex assay. Additionally, cortical brain tissue was analyzed from the delayed treated 5XFAD male mice. There were striking changes in multiple cytokines and chemokines across studies (Fig. 4). Notably, IL-6 and IL-22 were significantly reduced in plasma of male APPPS1-21 and 5XFAD mice, indicating a shift towards an anti-inflammatory milieu (Fig. 4a, c, d). The 5XFAD mice also exhibited a significant decrease in IL-1β, a proinflammatory cytokine, while simultaneously increasing IL-9, an anti-inflammatory cytokine, which reinforces the evidence that GV-971 reduces peripheral inflammation (Fig. 4c). Concurrently, cortical tissue from male 5XFAD mice had significant reductions in cytokines: IL-31 and chemokines: CCL3, CCL5, and Eotaxin (Fig. 4g). There were no significant changes in cytokine or chemokine levels in the female mice regardless of the experimental paradigm, thus further supporting a sex-specific effect of GV-971 (Fig. 4b, d). It is important to note that not all cytokines and chemokines analyzed were altered by GV-971 (Figure S6). These results indicate that GV-971 altered the peripheral immune system by shifting towards an anti-inflammatory profile, further suppressing inflammation and inflammatory signals in the brain.

### GV-971 treatment resulted in reduced plaque-localized Clec7a + disease-associated microglia and higher P2ry12^+^ homeostatic microglia in male mice only

**Fig. 5:**
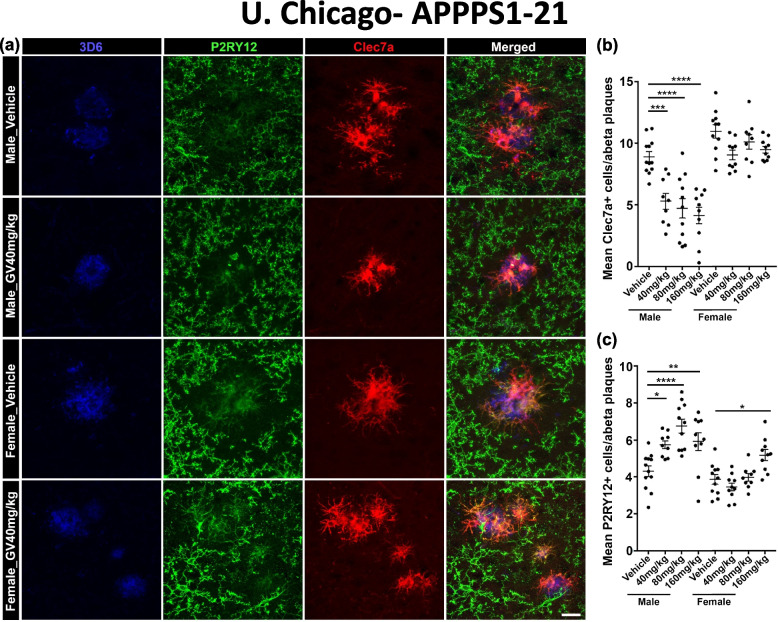
GV-971 significantly altered microglia inflammatory activation status surrounding Aβ plaques. **a** Representative immunofluorescent images of P2ry12^+^ microglia (green) and Clec7a^+^ microglia (red) clustering around 3D6^+^ Aβ plaque (blue) in mice from University of Chicago. **b** Quantification of the average number of cortical Clec7a^+^ cells within 0.02 mm2 area of 3D6^+^ Aβ plaque in cortices of APPPS1-21 mice treated with 40 mg/kg, 80 mg/kg, or 160 mg/kg GV-971 or vehicle (male *n* = 12–9, female *n* = 9–11). **c** Quantification of the average number of cortical P2ry12^+^ cells within 0.02 mm2 area of 3D6^+^ Aβ plaque in cortices of APPPS1-21 mice treated with 40 mg/kg, 80 mg/kg, or 160 mg/kg GV-971 or vehicle (male *n* = 12–9, female *n* = 9–11). Data are presented as mean SEM. Significance was determined using One-way ANOVA test followed by Tukey’s multiple comparison post hoc test. *, *P* < 0.05; **, *P* < 0.01; ***, *P* < 0.001; ****, *P* < 0.0001

Several studies have shown that microglia in degenerative conditions (MGnD) express Clec7a, or Dectin1, a pattern recognition receptor expressed by myeloid phagocytes, while microglia under homeostatic conditions express P2ry12, a chemoreceptor for ADP [53–55]. Specifically, Krasemann and colleagues established that Clec7a^+^ microglia were localized in close proximity to neuritic Aβ deposits while P2ry12^+^ microglia were not [55]. In our current study, we investigated Clec7a and P2ry12 expression patterns in conjunction with the Aβ-specific 3D6 antibody to elucidate the status of plaque-localized microglia by indirect immunofluorescence in APPPS1-21 mice (Fig. 3a: 3D6 blue, P2ry12 green, Clec7a red). We observed Clec7a^+^ microglia near 3D6^+^ amyloid plaques, while very few P2ry12^+^ microglia were found around 3D6^+^ plaques (Fig. 5a). 3-D Z-stacks allowed us to examine individual planes in all images to confirm and count plaque-localized Clec7a^+^ microglia. We found significant reductions in Clec7a^+^ plaque-localized microglia in male groups that were treated with 40 mg/kg, 80 mg/kg, and 160 mg/kg doses of GV-971 (Fig. 5a, b) without any apparent significant differences depending on GV dose. Female mice showed no significant reductions in Clec7a^+^ plaque-localized microglia (Fig. 5a, b). Furthermore, evaluation of homeostatic microglia in the 0.02mm^2^ area around the 3D6^+^ plaques showed higher P2ry12^+^ microglia in all-male groups treated with GV-971 (Fig. 5a, c). Similar to Clec7a^+^ plaque-localized microglia, GV-971 treated female mice showed no differences among P2ry12^+^ microglia number in either 40 mg/kg or 80 mg/kg groups; however, a significant increase was observed in 160 mg/kg group compared with control (Fig. 5a, c). These data lead us to conclude that GV-971 treatment significantly reduces microglia reactivity around Aβ positive plaques.

### GV-971 treatment reduced neuroinflammation in a sex-dependent manner in 9-month-old 5XFAD mice

**Fig. 6:**
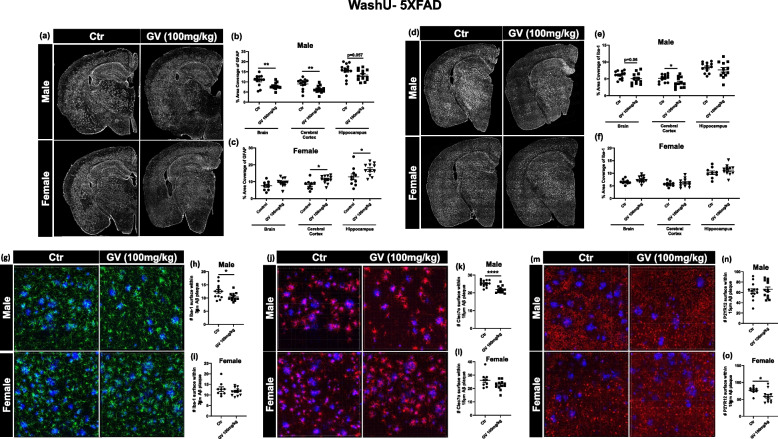
GV-971 significantly reduces glial inflammation in 9 month old 5XFAD male mice. **a** Representative images of brains stained for GFAP^+^ astrocytes in 5XFAD mice from Washington University in St. Louis. **b**, **c** Quantification of %area covered by GFAP^+^ astrocytes in whole brain, cortices and hippocampus of 5XFAD mice treated with 100 mg/kg GV-971 or vehicle (male = 13, female = 9–12). **d** Representative images of brains stained for Iba-1^+^ microglia. **e**, **f** Quantification of % area covered by Iba-1^+^ microglia in whole brain, cortices and hippocampus of 5XFAD mice treated with 100 mg/kg GV-971 or vehicle (male = 13, female = 9–12). **g** Representative images of Iba-1^+^ microglia (green) clustering around Aβ plaque (blue). **h**, **i** Quantification of the average number of Iba-1^+^ surface within 3 μM radius of Aβ plaque in cortices of 5XFAD mice treated with 100 mg/kg GV-971 or vehicle (male = 13, female = 9–12). **j** Representative images of Clec7a^+^ microglia (red) clustering around Aβ plaque (blue). **k**, **l** Quantification of the average number of Clec7a^+^ surface within 15 μM radius of Aβ plaque in cortices of 5XFAD mice treated with 100 mg/kg GV-971 or vehicle (male = 13, female = 9–12). **m** Representative images of P2ry12^+^ microglia (red) clustering around Aβ plaque (blue). **n**, **o** Quantification of the average number of P2ry12^+^ surface within 15 μM radius of Aβ plaque in cortices of 5XFAD mice treated with 100 mg/kg GV-971 or vehicle (male = 13, female = 9–12). Data presented as SEM. Significance determined using unpaired t-test (**d**). *, *P* < 0.05; **, *P* < 0.01; ***, *P* < 0.001; ****, *P* < 0.0001

It was previously reported that GV-971 has strong anti-inflammatory properties and reduced neuroinflammation [17]. To determine the effect GV-971 on neuroimmune cell profiles in the presence of robust Aβ burden, we probed delayed treated 5XFAD brain tissue for multiple inflammatory markers. Firstly, we investigated astrocyte activation and proliferation using glial fibrillary acidic protein (GFAP, a standard astrocyte marker). We observed that GV-971 significantly reduced GFAP immunostaining in multiple brain regions, including the cerebral cortex and hippocampus of only male mice (Fig. 6a, b). The inverse occurred in female mice treated with GV-971, with GFAP significantly increased (Fig. 6a, c). We determined that GV-971 profoundly impacted plaque-associated microglia reactivity, specifically reducing activated Clec7a^+^ microglia while increasing homeostatic P2ry12^+^ microglia in 3-month-old APPPS1-21 mice (Fig. 5). Therefore, we examined the effect GV-971 had on delayed treated 5XFAD mice by assessing Iba1^+^ microglia reactivity. We found that GV-971 significantly reduced Iba1 staining in the cerebral cortex of male mice, while there was no change in female Iba1 staining (Fig. 6d, e, f). We then analyzed peri-plaque microglial clustering and activation status by acquiring 3D images of microglia within 3-15 μm of an Aβ plaque and quantified by IMARIS software. Male mice treated with GV-971 had significantly fewer plaque-associated microglia (Fig. 6g, h). This finding was correlated with a significant reduction in Clec7a^+^ microglia surrounding Aβ plaques, denoting a decrease in microglial activation in only male mice (Fig. 6j, k). Female mice displayed no significant changes in the number of microglia nor Clec7a expression surrounding Aβplaques (Fig. 6g, i, j, l). There was no change found in P2ry12^+^ microglia surrounding plaques in male mice (Fig. 6m, n). However, there was a significant reduction in P2ry12^+^ microglia in female mice, indicating a reduction in homeostatic microglia (Fig. 6m, o). These results further support the sex effect, as GV-971 did not reduce Aβ plaque burden or neuroinflammation in female mice. It should be noted that while there was a significant reduction in Aβ plaque deposition following GV-971 treatment, there was still heavy plaque burden in the 9-month-old 5XFAD mice (Fig. 6e). This plaque reduction might not be enough to reverse microglia activation status. This data further supports the hypothesis that GV-971 significantly reduces neuroinflammation in a sex-dependent manner, specifically by altering plaque-associated microglia activation.

### GV-971 (160 mg/kg) treatment results in altered transcriptome in a sex-specific manner

**Fig. 7:**
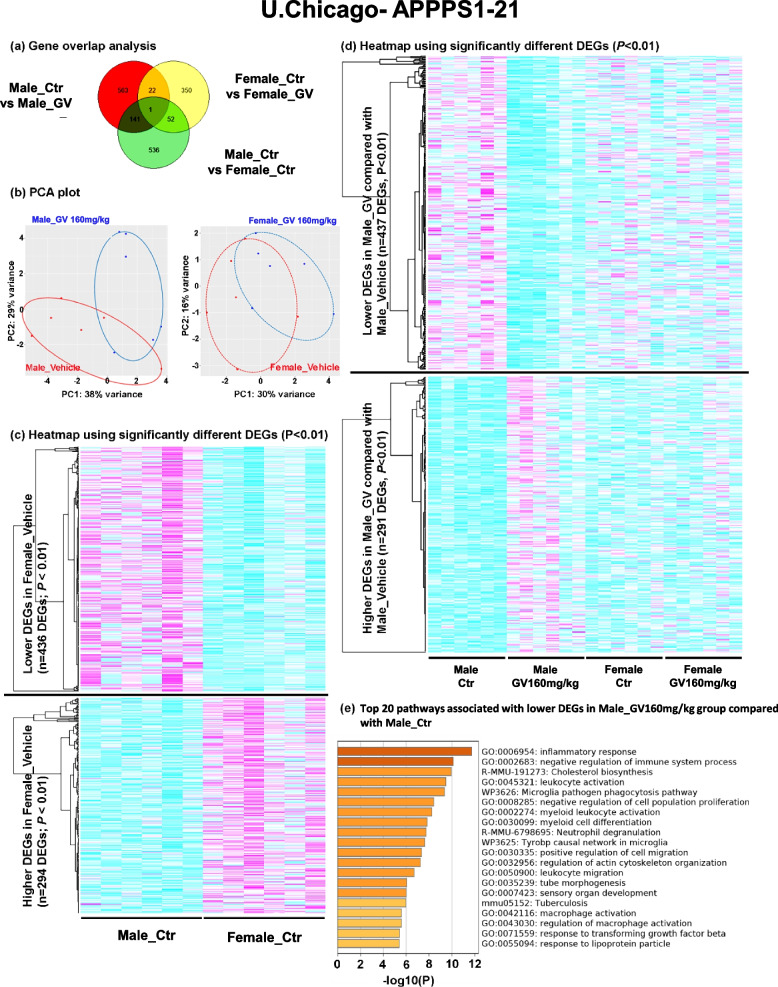
GV-971 treatment alters cerebral cortex transcriptome profiles in a sex-specific manner. **a** Gene overlap analysis demonstrating significant (*P* < 0.01) DEGs *n* = 728 in Male_Vehicle vs Male_GV, *n* = 426 DEGs in Female_Vehicle vs Female_GV and *n* = 730 DEGs in Male_Vehicle vs Female_Vehicle groups. Note only *n* = 23 DEGs that were common between GV-971-treated male and female groups compared with their respective vehicle-treated controls. **b** PCA plot. **c** Heatmap associated with lower DEGs (*n* = 436, *P* < 0.01) and higher DEGs (*n* = 294, *P* < 0.01) in Vehicle-treated female group compared with Vehicle-treated male group, note the sex-specific differences in DEGs between control male and female groups. Each column in an individual animal, *n* = 6 mice per group. **d** Heatmap associated with lower DEGs (*n* = 437, *P* < 0.01) and higher DEGs (*n* = 291, *P* < 0.01) in GV-971 treated male group compared with Vehicle-treated male group. Notice the female group columns exhibited no apparent differences for these genes. Each column is an individual animal, *n* = 6 mice per group. **e** GO biological processes, molecular functions, and KEGG pathways analysis based on the DEGs (*P* < 0.01) between vehicle-treated male and GV-971-treated male groups. Panel shows heatmap of top 20 pathways associated with lower DEGs in GV-971-treated male group compared with control

**Fig. 8:**
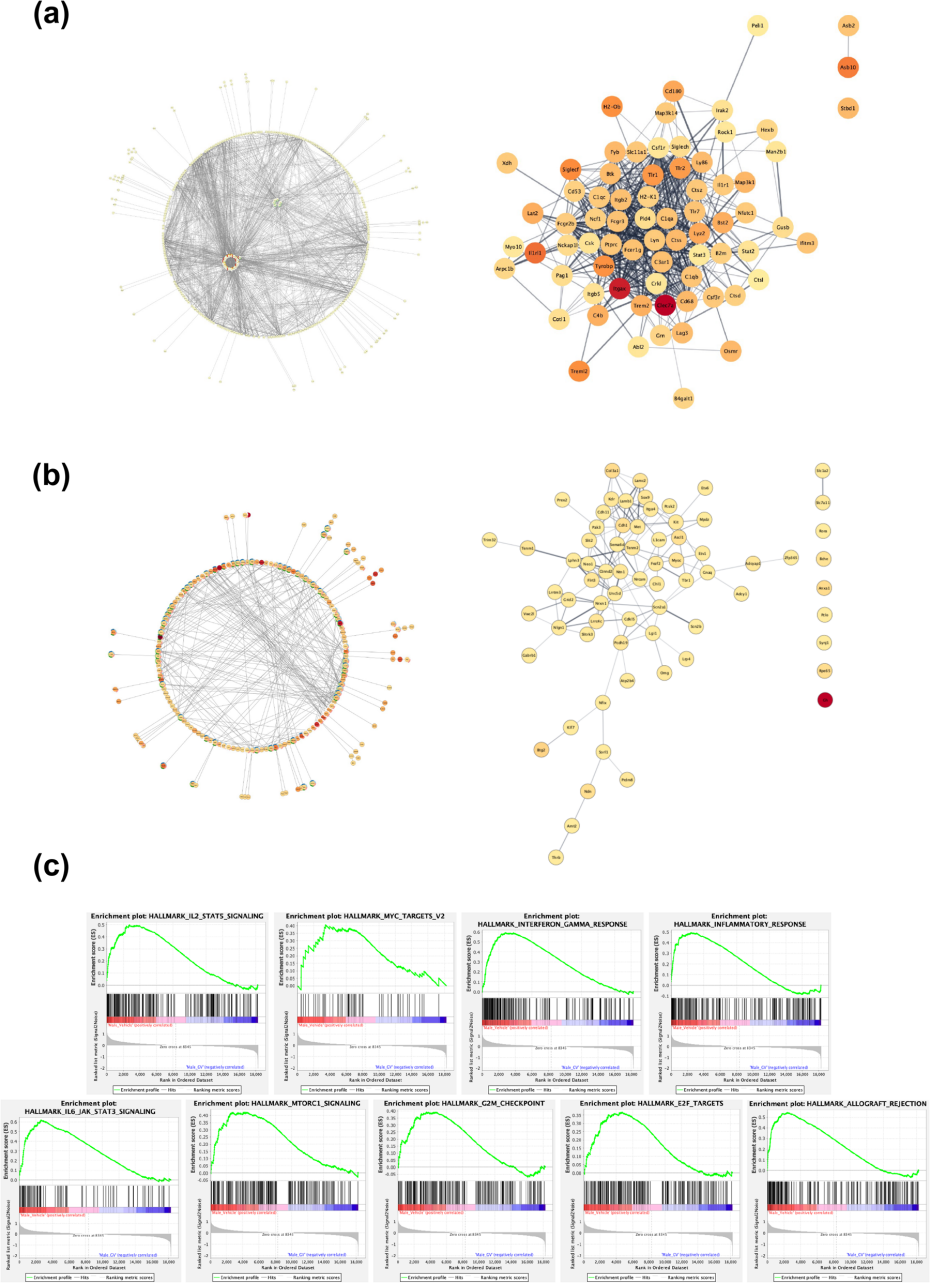
Cytoscape and GSEA analyses using significantly altered genes (*P* < 0.01) in GV-971 treated male group compared with control. **a** Cytoscape analysis identified immunity pathway as a major impacted pathway associated with lower DEGs (*P* < 0.01) in GV-971-treated male compared with control. Most enriched genes are highlighted in darker shade. **b** Cytoscape analysis identified neuronal development pathway as a major impacted pathway associated with higher DEGs (*P* < 0.01) in GV-971 treated group compared with control. Most enriched genes are highlighted in darker shade. **c** GSEA enrichment results showed 9 significantly enriched gene sets out of 44 upregulated gene sets in Vehicle- treated male group compared with GV-971-treated male group

We performed RNAseq to examine transcriptional changes in the cerebral cortex of animals treated with GV-971. Bulk RNA was extracted from the dorsal cerebral cortex of male and female APPPS1-21 mice subject to vehicle or 160 mg/kg GV-971 treatment. Here, we chose only GV-971 160 mg/kg groups as these groups showed significant differences in Aβ burden (Fig. 1) and Clec7a^+^ microglia status (Fig. 5) in a sex-specific manner. For RNAseq comparisons, we chose *P* < 0.01 as a significant differences for further investigations (Fig. 7). We observed a total of 728 DEGs altered in the male vehicle compared with the GV-treatment group, and only 426 DEGs altered in the female vehicle compared with the GV-treatment group. However, gene overlap analysis showed only 23 genes common between GV-971-treated male and female groups compared with their respective vehicle-treated controls (Fig. 7a), thus suggesting a sex-specific effect of GV-971 on cerebral cortex transcriptomes. PCA plot analysis showed differences in transcriptome profiles with GV-971 treatments (Fig. 7b); specifically, male_GV-971 showed a more pronounced shift in their clusters compared with female_GV-971 with respect to their vehicle-treated controls. Interestingly, we observed marked differences in gene expression between vehicle-treated male and female groups (*n* = 730 DEGs; 436 DEGs downregulated and 294 DEGs upregulated in females; *P* < 0.01; Fig. 7a, c). For the subsequent comparisons, we focused on the male group transcriptome changes as these groups showed profound GV-971-dependent effects on Aβ amyloidosis. We found 437 DEGs downregulated and 291 DEGs upregulated in GV-971-treated male groups compared with vehicle-treated animals (Fig. 7d). Interestingly, none of these DEGs were significantly altered similarly in female groups with the GV-971 treatment (Fig. 7d). We then extracted these upregulated and downregulated genes for pathway analysis and network string analysis or gene set enrichment analyses using Metascape (Fig. 7e), Cytoscape (Fig. 8a, b), and GSEA analyses (Fig. 8c) respectively. Metascape analysis of the 437 downregulated DEGs showed several pathways that were associated with inflammation and microglia activities such as inflammatory responses (GO:0006954), negative regulation of immune system process (GO:0002683), microglia pathogen phagocytosis pathway (WP3626), myeloid cell differentiation (GO:0030099), Tyrobp causal network in microglia (WP3625), macrophage activation (GO:0042116) and response to transforming growth factor-beta (TGF-beta) as shown in Fig. 7e. These pathways associated with downregulated DEGs suggest that GV-971 160 mg/kg treatment reduced inflammation and altered activity in microglia phagocytosis. Furthermore, we used Cytoscape analysis to perform network string analysis and identify potential gene targets in this model. Using the list of downregulated genes in the male GV-971 160 mg/kg group compared with vehicle-treated controls (Fig. 8a), Cytoscape identified immune activity-related pathways as the major impacted network. Here, several genes that are associated with the complement system (C1qa, C1qb, C1qc, C3ar) and phagocytic processes (TLR1, TLR2, TLR7, CD68, clec7a, Tyrobp) were identified. Interestingly, analysis of up-regulated genes in the male group GV-160 mg/kg group compared with vehicle-treated controls revealed neuronal development as the principal impacted pathway (Fig. 8b). Similar to Metascape and Cytoscape, GSEA analysis identified 44 out of 50 gene sets that were upregulated in vehicle-treated males compared with GV-971 160 mg/kg treated male groups. Amongst the 9 gene sets that were significantly enriched at a nominal *p*-value of < 0.05, IL2-STAT signaling, interferon-gamma response, inflammatory response, IL6-JAK-STAT3 signaling, MTORC1 signaling were signaling pathways that reflect lower inflammatory responses in GV-971-treated male groups (Fig. 8c). None of these inflammatory pathways or microglia-related complement/phagocytosis were highlighted among any other group comparisons.

To further corroborate our RNAseq dataset, we extended the analysis to examine gene expression across all doses of GV-971. For this, we performed qPCR analysis of RNA extracted from the dorsal cortices of frozen brain hemispheres to assess expression levels of genes related to inflammation, microglia, and neurodevelopment. In males, complement system-related gene C3ar1 expression was significantly reduced in the GV 160 mg/kg group compared with the GV 40 mg/kg group (Figure S7). Furthermore, male mice showed a trend in dose reduction in Clec7a expression, with the GV 160 mg/kg group having a significant reduction. Male mice treated with 40 mg/kg or 80 mg/kg of GV-971 have increased neuronally expressed cadherin 1 (Cdh1) expression compared with vehicle-treated males. However, males treated with 160 mg/kg of GV-971 did not have significantly different expression levels of Cdh1 compared with vehicle-treated males (Figure S6). No other targets showed significant differences between groups for male mice. In female mice, only the neurodevelopment-related Cdh1 expression was significantly increased in the GV 80 mg/kg group compared with control females. The female GV 80 mg/kg group also had significantly higher levels of Cdh1 expression than the 160 mg/kg GV group. No other targets showed significantly altered expression between female groups (Figure S7).

The RNAseq data revealed that GV-971 downregulated multiple inflammatory genes associated with microglial activation, complement pathways, and phagocytosis. Therefore, to investigate these findings in the delayed treated 5XFAD mice, we performed targeted qPCR analysis on cortical tissue using Fluidigm Biomark HD. Similar to the APPPS1-21 mice, GV-971 significantly downregulated inflammatory genes, specifically Hexb, a disease-associated microglia marker, Itgam, a complement receptor, and IL-1b, a pro-inflammatory cytokine. These changes were only found in male mice treated with GV-971, further indicating that GV-971 functions in a sex-dependent manner (Fig. S8). Collectively, these extensive dataset analyses show that GV-971 treatment resulted in lower inflammation and altered microglia phagocytosis activities only in male groups.

## Discussion

In a series of preceding efforts, we established a critical role of microbiota in cerebral amyloidosis and microglial physiology. To investigate whether the gut microbiome is a novel therapeutic target with treatments beyond antibiotics, we investigated the efficacy of sodium oligomannate (GV-971) in modulation of Aβ amyloidosis and neuroinflammation. Wang et al. revealed that in the 5xFAD transgenic model of Aβ amyloidosis, sodium oligomannate, or GV-971, reduced Aβ amyloidosis that correlated with alterations in the gut microbiota [17]. The mechanism proposed was that dysbiosis associated with aging in the 5XFAD transgenic mouse model was a driving factor in promoting Th1/M1 microglia- predominant neuroinflammation in AD progression. GV-971 treatment resulted in the reconditioning of gut microbiota and metabolic functions to reduce peripheral and neuroinflammatory responses as well as improve cognitive behaviors. To confirm and extend these findings, we treated APPPS1-21 mice and 5XFAD mice, two established mouse models of Aβ amyloidosis, with GV-971. Male and female APPPS1-21 mice, at the onset of amyloid deposition, were treated and analyzed at The University of Chicago while 5XFAD mice with a high level of amyloid deposition were treated and analyzed at Washington University, St. Louis. Notably, these latter experiments were performed independently at the host institutions without knowledge of each other’s experiments until after they had been completed. Thus, we made no attempts to coordinate the study protocols or experimental design during the course of these studies. Remarkably, our collective dataset has allowed us to offer several important insights pertaining to the role of GV-971 in altering the gut microbiota that is correlated with altered metabolism and reductions of peripheral inflammation, reductions in neuroinflammation and Aβ amyloidosis. Importantly, we now identify a sex-specific effect that was not reported by Wang et al. [17].

First, GV-971 treatment results in male-specific reductions in brain Aβ deposition. Second, microbiota analysis shows sex-specific microbiota profiles in vehicle-treated male and female groups that are impacted in a sex-specific manner by GV-971 treatment. The changes in microbiota composition translated to alterations in bacterial metabolism as well as reductions in peripheral inflammatory markers. Third, analysis of microglia activation state using the activation marker Clec7a and homeostatic marker P2ry12 showed significant reductions in Clec7a^+^ microglia only in GV-971-treated male groups, results that were further corroborated by analysis of cerebral cortical transcriptomes. Collectively, our studies mirrors and thus strengthens our previous findings that antibiotic-mediated perturbations of the gut microbiota plays an important role in sex-specific modulation of Aβ amyloidosis.

Communication between the gut microbiome and microglia has been proposed to be mediated through signal transduction via the vagus nerve or gut-derived cytokines and metabolites in the peripheral circulation [7]. Studies utilizing germ-free (GF) mice have shown that the absence of microbiota results in the cessation of microglial development, as evidenced by immature phenotype and high levels of expression of genes characteristic of early and pre-microglia in adult mice [45, 53, 56]. Furthermore, gut microbiota composition has been demonstrated to be an important factor in regulating microglial maturation and function in a sex-specific manner [53, 57]. Our previous work also established that ABX-induced perturbations of gut microbiota are associated with sex-specific alterations in microglial phenotypes [15, 19, 58]. Prior investigations of 5xFAD mice treated with GV-971 revealed that this agent shifted microbiome profiles and was associated with a decrease in phagocytosis and microglial activation [17]. In the current study, we also showed that GV-971 treatment influenced microglial phenotypes in both the APPPS1-21 and 5XFAD mouse models. Specifically, we demonstrated that GV-971 reduced plaque-associated Clec7a^+^ activated microglia and increased P2ry12^+^ homeostatic microglia, a phenomenon that was specific to male mice. We also found that GV-971 significantly reduced GFAP expression in male 5XFAD mice, suggesting a decrease in astrocyte reactivity. To our knowledge, this is the first report of GV-971 impacting Aβ amyloidosis, astrocyte activation, and microglial activation state and phenotypes in a sex-specific manner.

Previously published work by Wang et al. [17] explored the beneficial behavioral effects of GV-971 treatment on APP/PS1 mice as well as physiological effects on 5xFAD mice. In contrast, our studies have focused on the assessment of amyloid burden, glia-associated inflammation, and cortical transcriptional analyses to further understand the effectiveness of GV-971 in independent mouse models treated at multiple stages of disease. We have shown that in APPPS1-21 mice treated in the early stage of disease (2–3 months of age) and 5X FAD mice treated at a late stage of amyloidosis (7–9 months of age), GV-971 alters the gut microbiota and attenuates Aβ deposition in brains of the two models. As maximal effects of amyloid lowering were found in APPPS1-21 mice at the lowest dose utilized (40 mg/kg), further studies are warranted to assess effects at lower doses. We found that GV-971 altered β diversity in the APPPS1-21 mice as well as significantly modifying bacterial species abundance at both time points. What is most compelling was that despite the studies being conducted at separate universities/mouse facilities, GV-971 altered multiple overlapping bacterial species in APPPS1-21 and 5XFAD mice studies. For example, GV-971 significantly reduced Desulfovibrio (phylum Protobacteria) while increasing Bifidobacterium pseudolongum (phylum Actinobacteria), a common probiotic bacteria [59] in both the APPPS1-21 and 5XFAD mouse models (Table 1). Gut dysbiosis strongly correlates with irregular immune responses accompanied by increased production of inflammatory cytokines [51]. Our study found that GV-971 significantly reduced pro-inflammatory cytokines in both the periphery as well as in cortical tissues, specifically reducing IL-6, IL-22, CCL5, and CCL3 which are known to regulate T-cell mediated inflammation. Interestingly, it has been reported that microbiota-derived tryptophan as well as other metabolites mediates immune cell responses and cytokine production [50, 60]. Tryptophan plays a pivotal role in regulating both CNS and immune functions. Serving as the precursor to serotonin, a critical neurotransmitter, it also contributes to the regulation of the enteric system. Importantly, an imbalance of tryptophan metabolism and downstream pathways have been implicated in multiple neurodegenerative and neuropsychiatric diseases [61]. We report that GV-971 significantly increased amino acid and tryptophan metabolism in male 5XFAD mice, further supporting the hypothesis that the compound corrected microbiome dysbiosis, and altered metabolism resulting in reduced peripheral inflammation and neuroinflammation.

Our results are the first to suggest that GV-971-mediated reduction of Aβ burden is driven in a sex-specific manner. This is a critical finding as GV-971 has approval for clinical administration in China as well as clinical trials within the United States (NCT04520412). The reasons for the relative sparing in female animals are likely complex and could involve roles for ovarian hormones, differences in gut microbiota profiles compared with males [62], and differences in transcriptional outcomes in microglia [44, 63]. In this regard, our transcriptome data reveal that males treated with the highest dose of the GV-971 compound have lower levels of pro-inflammatory markers. Surprisingly, our data also suggests that the genes responsible for neuronal development e.g., Cdh1 and Cdh11 [64–66] are upregulated in male and female APPPS1-21 mice treated with GV-971.

While it might be argued that GV-971-mediated alterations in microbiota, microglial phenotypes, and male-specific reductions in Aβ deposition support a model that involves the microbiota-microglia-amyloidosis axis, there are caveats. Of these, GV-971, a mixture of oligosaccharides with a degree of polymerization (dp) from 2 to 10 [67] was shown to be present in the cerebral spinal fluid following intraperitoneal injection, but information pertaining to the levels present in the brain are not known but will be critical as studies have demonstrated that this agent binds to diverse Aβ species in vitro*,* inhibits Aβ fibril formation and destabilizes preformed fibrils into non-toxic monomers [17]. Future GC-LCMS studies to investigate the presence or absence of GV-971 in the brain will be critical to address this issue. This caveat notwithstanding, our findings provide compelling evidence that in males, GV-971 reduces Aβ deposition and plaque-associated reactive microglia. Specifically, in 5X FAD mice, we found that GV-971 reduced Aβ plaque halo which contains non-fibrillar Aβ protein [68]. Based on our findings, we hypothesize that GV-971 alters microglial activity in a manner leading to phagocytosis of the plaque halo, thus reducing amyloid burden and subsequent disease pathology. The role(s) of specific microbial species and metabolites generated by microbes (or host) in driving GV-971-mediated alterations in Aβ amyloidosis and microglial function is clearly warranted and will be the focus of future studies.

## Supplementary Information

**Additional file 1: Supplemental Figure 1.** Gut microbiome composition differs significantly between University of Chicago and Washington University in St. Louis. Analysis of bacterial α-diversity and β-diversity in fecal content from 9-week-old APPPS1-21 male mice collected at the University of Chicago and Washington University in St. Louis. (a) Shannon index, (b) Pielou species evenness. (d) PCoA plot generated by using unweighted unifrac distance metric. Diversity analyses, including alpha and beta diversity, alpha rarefaction, and group significance were analyzed by QIIME and QIIME2. Data are presented as mean SEM. Significance was determined using Two-way ANOVA . *, *P* < 0.05; **, *P* < 0.01; ***, *P* < 0.001; ****, *P* < 0.0001. **Supplemental Figure 2.** GV-971 targets Aβ plaque halo in a sex-dependent manner. (a) Representative immunofluorescent images of HJ3.4^+^ Aβ (red) surrounding X34^+^ Aβ (blue). White * indicates regions of reduced plaque halo. (b,c) Quantification of an average number of HJ3.4 ^+^ Aβ surfaces within 5μM X34^+^ Aβ surface plaque in cortices of 5XFAD mice treated with 100mg/kg GV-971 or vehicle (male = 13, female = 9-12). Data are presented as mean SEM. Significance was determined using unpaired t-test (d). *, *P* < 0.05; **, *P* < 0.01; ***, *P* < 0.001; ****, *P* < 0.0001. **Supplemental Figure 3.** GV-971 alters amino acid metabolism. GC-nCI-MS and PFBBR derivtization heatmap analysis of metabolite abundance in cecal content from 5XFAD mice treated with 100mg/kg GV-971 or vehicle (male = 13, female 9-12). **Supplemental Figure 4.** GV-971 modifies tryptophan metabolism. LCMS/MS heatmap analysis of tryptophan pathway, indole pathway, and kynurenine pathway metabolite concentrations in cecal content from 5XFAD mice treated with 100mg/kg GV-971 or vehicle (male = 13, female 9-12). **Supplemental Figure 5.** GV-971 influences primary and secondary bile acid metabolism. LCMS/MS heatmap analysis of primary and secondary bile acid concentrations in cecal content from 5XFAD mice treated with 100mg/kg GV-971 or vehicle (male = 13, female 9-12). **Supplemental Figure 6.** GV-971 Significantly change peripheral and neuro-cytokine and chemokine production. Pie chart denoting the distribution of cytokine/chemokine production following GV-971 treatment. Chart is characterized into four groups based on expression levels compared to the control groups: Increased, Decreased, Not significant, and Non Detected. (a). Serum analyzed from the University of Chicago APPPS1-21 male and female mice treated with 160mg/kg GV-971. (b). Serum analyzed from Washington University 5XFAD male and female mice treated with 100mg/kg GV-971. (c). Cortical tissue analyzed from Washington University 5XFAD male mice treated with 100mg/kg GV-97. **Supplemental Figure 7.** GV-971 significantly alters microglia activation and neurodevelopment gene expression. (a). Quantitative PCR analysis of inflammatory, microglial, and neurodevelopment gene expression from bulk cortical tissue of male APPPS1-21 mice treated with GV-971 or vehicle. (b) Quantitative PCR analysis of inflammatory, microglial, and neurodevelopment gene expression from bulk cortical tissue of female APPPS1-21 mice treated with GV-971 or vehicle. Data are presented as mean SEM. Significance was determined using 2 way ANOVA followed by post hoc Tukey’s multiple comparisons. *, *P* < 0.05; **, *P* < 0.01; ***, *P* < 0.001; ****, *P* < 0.0001. **Supplemental Figure 8.** GV-971 alters inflammatory markers in 9 month old 5XFAD male mice. Heat map analysis of bulk RNA in cortices of 5XFAD mice following 100mg/kg GV-971 or vehicle treatment (A) male mice *n*=13, (B) female mice *n*= 9-12. Graph generated by hierarchical gene clustering based on groups. Statistical analyses were performed using an unpaired t- test. *, *P* < 0.05.

## Data Availability

All data are available in the main text or the supplementary materials.
