# Correction: Sodium oligomannate alters gut microbiota, reduces cerebral amyloidosis and reactive microglia in a sex-specific manner

**DOI:** 10.1186/s13024-024-00764-2

**Published:** 2024-10-10

**Authors:** Megan E. Bosch, Hemraj B. Dodiya, Julia Michalkiewicz, Choonghee Lee, Shabana M. Shaik, Ian Q. Weigle, Can Zhang, Jack Osborn, Aishwarya Nambiar, Priyam Patel, Samira Parhizkar, Xiaoqiong Zhang, Marie L. Laury, Prasenjit Mondal, Ashley Gomm, Matthew John Schipma, Dania Mallah, Oleg Butovsky, Eugene B. Chang, Rudolph E. Tanzi, Jack A. Gilbert, David M. Holtzman, Sangram S. Sisodia

**Affiliations:** 1 Department of Neurology, Hope Center for Neurological Disorders, Knight Alzheimer’s Disease Research Center, Washington University in St. Louis, St. Louis, USA; 2 Department of Neurobiology, University of Chicago, Chicago, USA; 3 Genetics and Aging Research Unit, McCance Center for Brain Health, MassGeneral Institute for Neurodegenerative Disease, Department of Neurology, Massachusetts General Hospital, Harvard Medical School, Boston, MA USA; 4 Center for Genetic Medicine, Northwestern University, Chicago, USA; 5 Genome Technology Access Center, Washington University in St. Louis, St. Louis, USA; 6 Ann Romney Center for Neurologic Diseases, Department of Neurology, Brigham and Women’s Hospital, Harvard Medical School, Boston, MA USA; 7 Department Medicine, Section of Gastroenterology, Hepatology, and Nutrition, The University of Chicago, Chicago, USA; 8 Department of Pediatrics and Scripps Institution of Oceanography, UCSD, San Diego, USA

Molecular Neurodegeneration (2024) 19:18

10.1186/s13024-023-00700-w

The original article erroneously presents incorrect graph labels in the caption of Fig. 4. The corrected Fig. 4 caption alongside its respective figure can be viewed ahead in this Correction article.

**Fig. 4:**
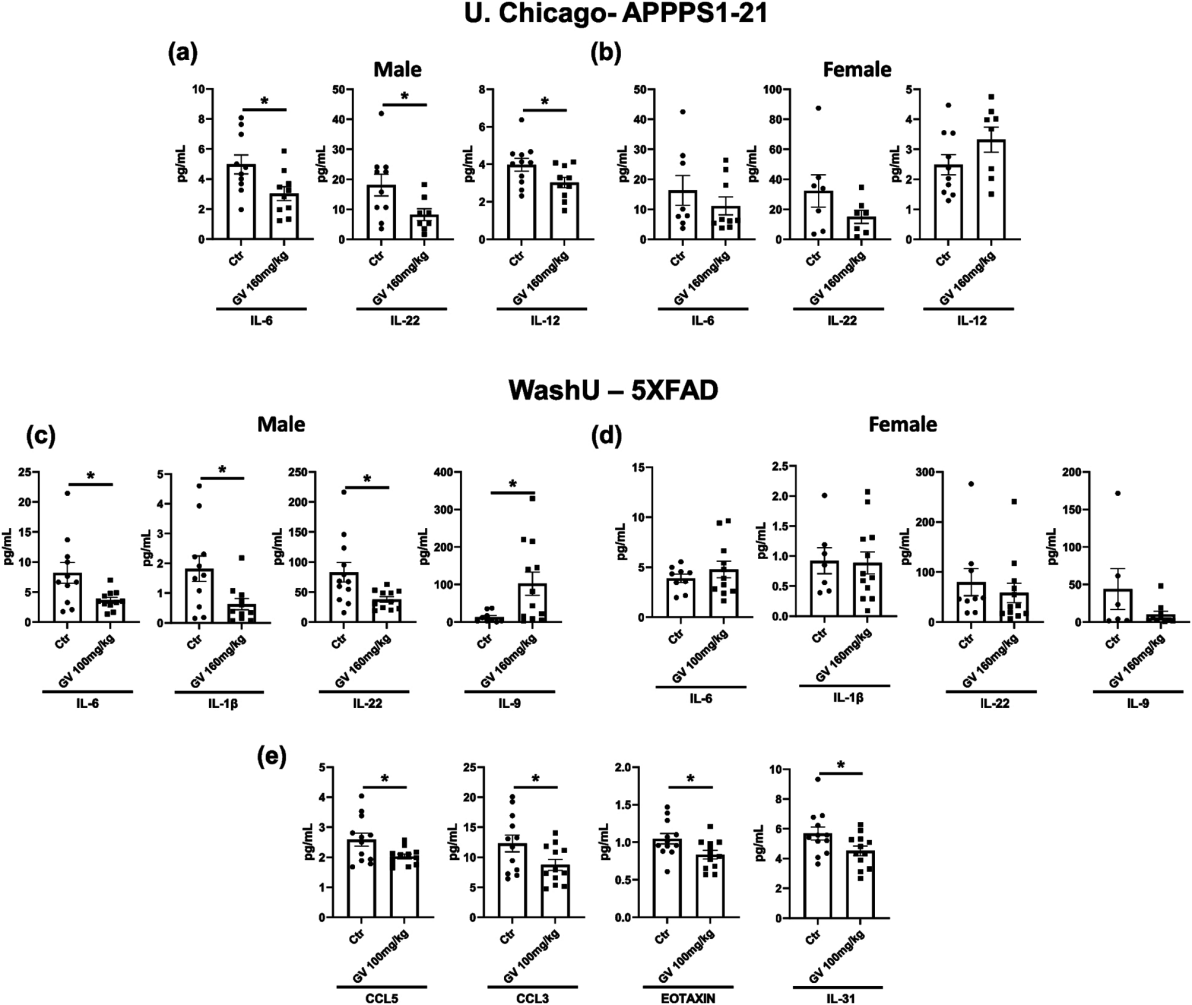
GV-971 modifies cytokine and chemokine levels in peripheral blood and cortical tissues. (**a**) Quantification of cytokine and chemokine concentrations in the serum of APPPS1-21 male mice treated with 160mg/kg GV-971 or vehicle from the University of Chicago (n = 10–11). (**b**) Quantification of cytokine and chemokine concentrations in the serum of APPPS1-21 female mice treated with 160mg/kg GV-971 or vehicle (n = 8–10). (**c**) Quantification of cytokine and chemokine concentrations in the serum of 5XFAD male mice treated with 100mg/kg GV-971 or vehicle from Washington University in St. Louis (n = 12–13). (**d**) Quantification of cytokine and chemokine concentrations in the serum of 5XFAD female mice treated with 100mg/kg GV-971 or vehicle (n = 9–12). (**e**) Quantification of cytokine and chemokine concentrations in the cortical tissue of 5XFAD male mice treated with 100mg/kg GV-971 or vehicle (n = 12–13). Data presented as SEM. Significance determined using unpaired t-test. *, P<0.05; **, P<0.01; ***, P<0.001; ****, P<0.0001

