# Supplementary material for: Sodium oligomannate alters gut microbiota, reduces cerebral amyloidosis and reactive microglia in a sex-specific manner
Source: Mol Neurodegener. 2024 Feb 17;19:18. doi: 10.1186/s13024-023-00700-w (PMC10874048; doi:10.1186/s13024-023-00700-w)
Supplement: Supplementary file 1 — Additional file 1: Supplemental Figure 1. Gut microbiome composition differs significantly between University of Chicago and Washington University in St. Louis. Analysis of bacterial α-diversity and β-diversity in fecal content from 9-week-old APPPS1-21 male mice collected at the University of Chicago and Washington University in St. Louis. (a) Shannon index, (b) Pielou species evenness. (d) PCoA plot generated by using unweighted unifrac distance metric. Diversity analyses, including alpha and beta diversity, alpha rarefaction, and group significance were analyzed by QIIME and QIIME2. Data are presented as mean SEM. Significance was determined using Two-way ANOVA . *, P < 0.05; **, P < 0.01; ***, P < 0.001; ****, P < 0.0001. Supplemental Figure 2. GV-971 targets Aβ plaque halo in a sex-dependent manner. (a) Representative immunofluorescent images of HJ3.4+ Aβ (red) surrounding X34+ Aβ (blue). White * indicates regions of reduced plaque halo. (b,c) Quantification of an average number of HJ3.4 + Aβ surfaces within 5μM X34+ Aβ surface plaque in cortices of 5XFAD mice treated with 100mg/kg GV-971 or vehicle (male = 13, female = 9-12). Data are presented as mean SEM. Significance was determined using unpaired t-test (d). *, P < 0.05; **, P < 0.01; ***, P < 0.001; ****, P < 0.0001. Supplemental Figure 3. GV-971 alters amino acid metabolism. GC-nCI-MS and PFBBR derivtization heatmap analysis of metabolite abundance in cecal content from 5XFAD mice treated with 100mg/kg GV-971 or vehicle (male = 13, female 9-12). Supplemental Figure 4. GV-971 modifies tryptophan metabolism. LCMS/MS heatmap analysis of tryptophan pathway, indole pathway, and kynurenine pathway metabolite concentrations in cecal content from 5XFAD mice treated with 100mg/kg GV-971 or vehicle (male = 13, female 9-12). Supplemental Figure 5. GV-971 influences primary and secondary bile acid metabolism. LCMS/MS heatmap analysis of primary and secondary bile acid concentrations in cecal content from 5XFAD mice t [file 13024_2023_700_MOESM1_ESM.zip › Additional file 1.pdf]

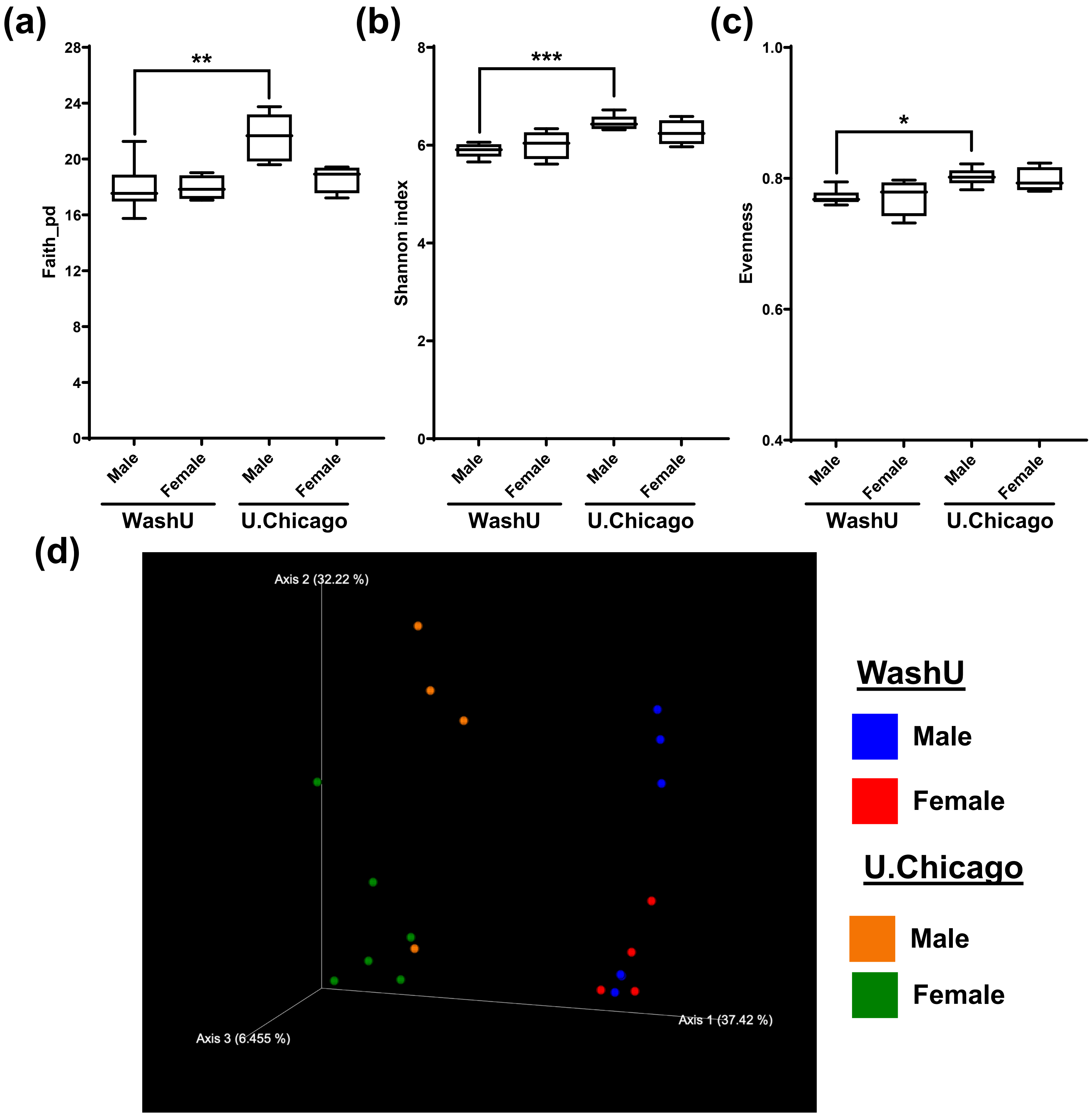

(a)
Faith_pd
28
24
20
16
12
8
4
0
**
Male
Female
Male
Female
WashU
U.Chicago
(b)
Shannon index
8
6
4
2
0
***
Male
Female
Male
Female
WashU
U.Chicago
(c)
Evenness
1.0
0.8
0.6
0.4
*
Male
Female
Male
Female
WashU
U.Chicago
(d)
Axis 2 (32.22 %)
Axis 1 (37.42 %)
Axis 3 (6.455 %)
WashU
Male
Female
U.Chicago
Male
Female

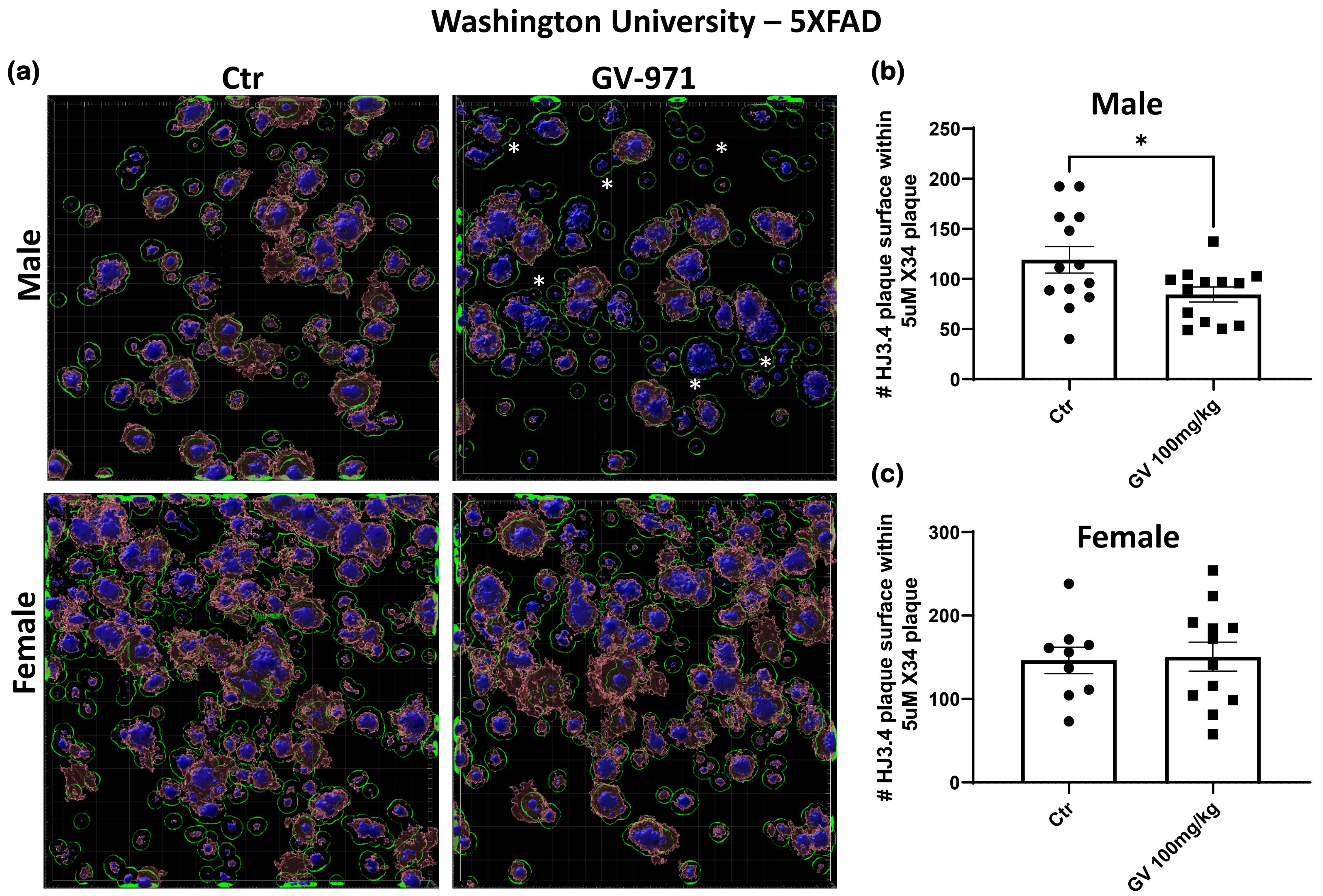

Washington University – 5XFAD
(a)
Ctr
GV-971
Male
Female
(b)
Male
HJ3.4 plaque surface within 5uM X34 plaque
250
200
150
100
50
0
*
Ctr
GV 100mg/kg
(c)
Female
HJ3.4 plaque surface within 5uM X34 plaque
300
200
100
0
Ctr
GV 100mg/kg

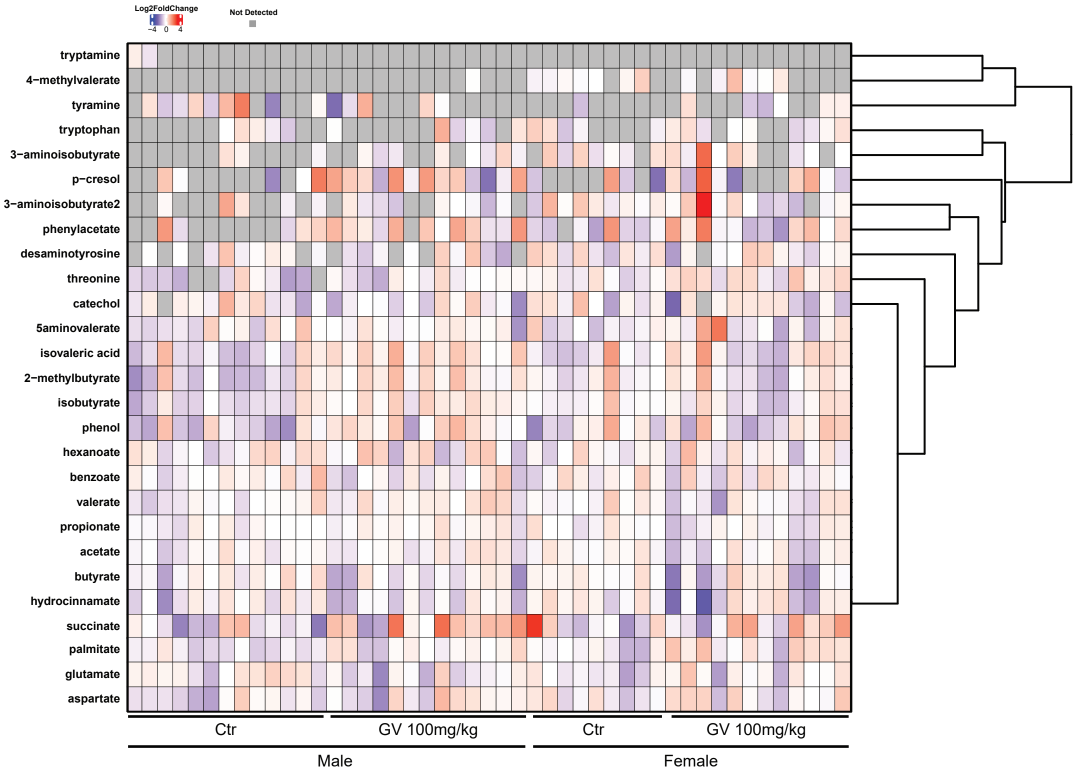

Log2FoldChange
−4 0 4
Not Detected
tryptamine
4−methylvalerate
tyramine
tryptophan
3−aminoisobutyrate
p−cresol
3−aminoisobutyrate2
phenylacetate
desaminotyrosine
threonine
catechol
5aminovalerate
isovaleric acid
2−methylbutyrate
isobutyrate
phenol
hexanoate
benzoate
valerate
propionate
acetate
butyrate
hydrocinnamate
succinate
palmitate
glutamate
aspartate
Ctr
GV 100mg/kg
Ctr
GV 100mg/kg
Male
Female

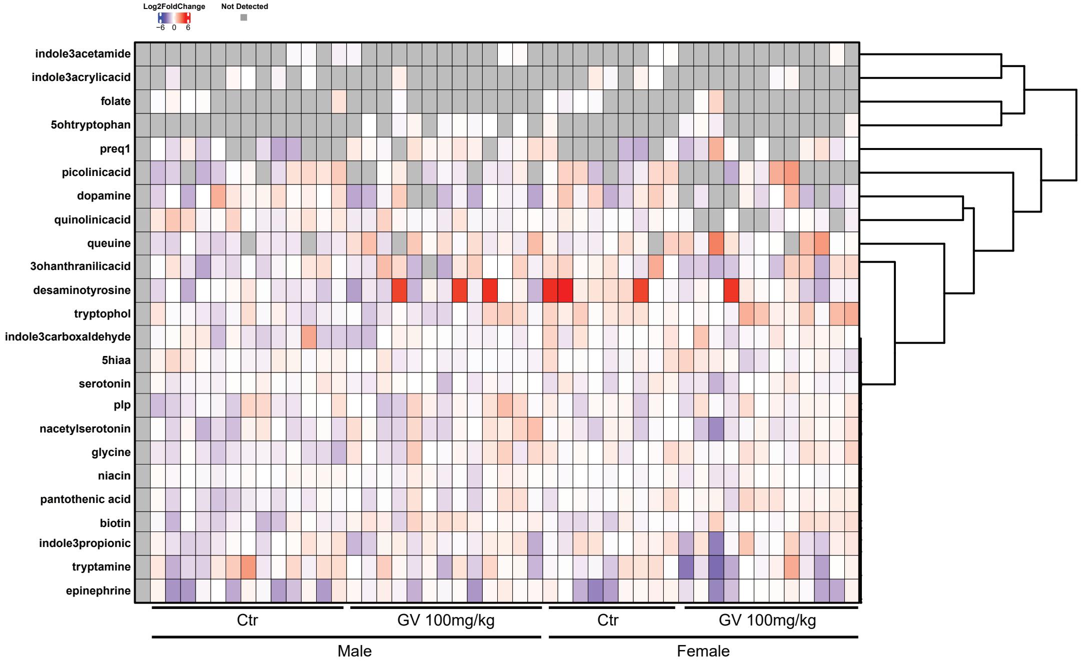

Log2FoldChange
−6 0 6
Not Detected
indole3acetamide
indole3acrylicacid
folate
5ohtryptophan
preq1
picolinicacid
dopamine
quinolinicacid
queuine
3ohanthranilicacid
desaminotyrosine
tryptophol
indole3carboxaldehyde
5hiaa
serotonin
plp
nacetylserotonin
glycine
niacin
pantothenic acid
biotin
indole3propionic
tryptamine
epinephrine
Ctr
GV 100mg/kg
Ctr
GV 100mg/kg
Male
Female

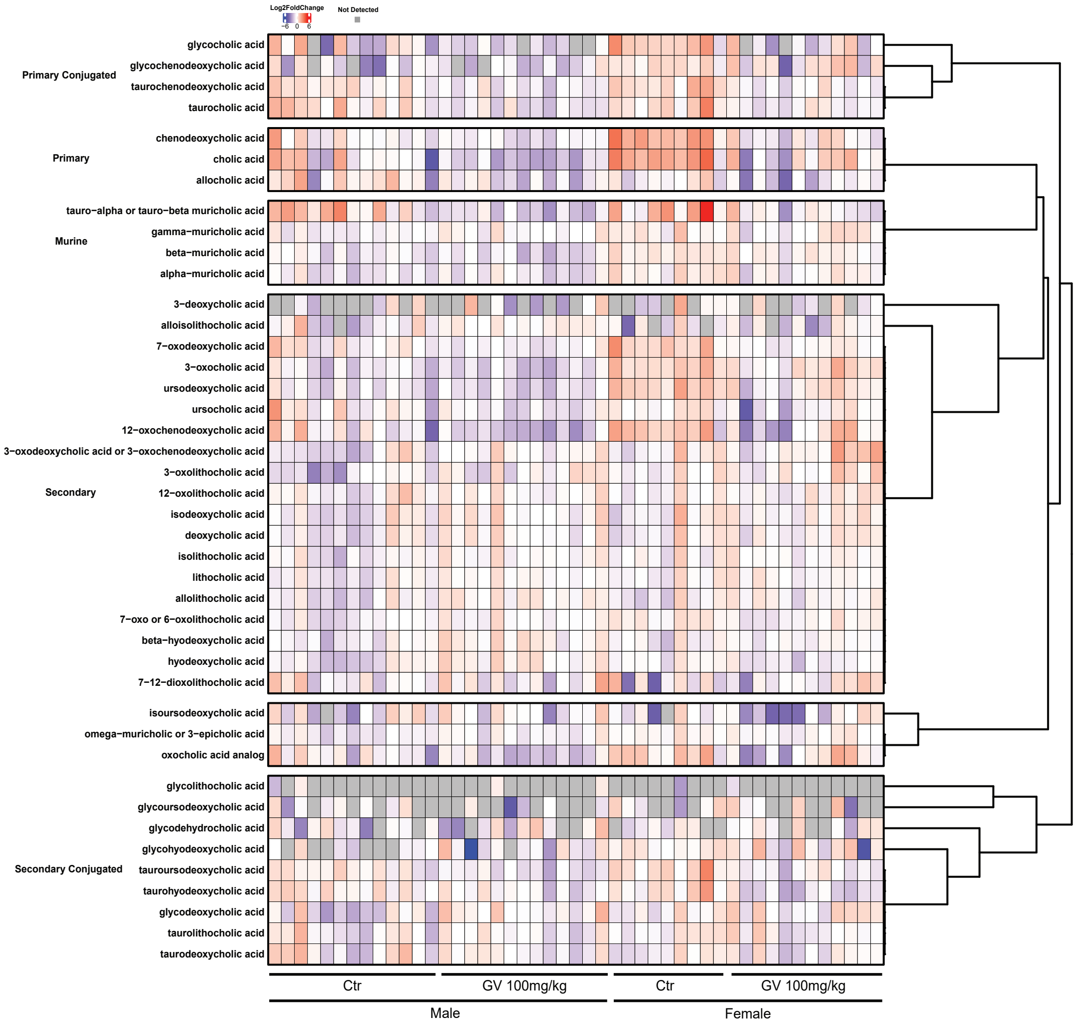

Log2FoldChange
−6
0
6
Not Detected
Primary Conjugated
glycocholic acid
glycochenodeoxycholic acid
taurochenodeoxycholic acid
taurocholic acid
Primary
chenodeoxycholic acid
cholic acid
allocholic acid
Murine
tauro−alpha or tauro−beta muricholic acid
gamma−muricholic acid
beta−muricholic acid
alpha−muricholic acid
Secondary
3−deoxycholic acid
alloisolithocholic acid
7−oxodeoxycholic acid
3−oxocholic acid
ursodeoxycholic acid
ursocholic acid
12−oxochenodeoxycholic acid
3−oxodeoxycholic acid or 3−oxochenodeoxycholic acid
3−oxolithocholic acid
12−oxolithocholic acid
isodeoxycholic acid
deoxycholic acid
isolithocholic acid
lithocholic acid
allolithocholic acid
7−oxo or 6−oxolithocholic acid
beta−hyodeoxycholic acid
hyodeoxycholic acid
7−12−dioxolithocholic acid
isoursodeoxycholic acid
omega−muricholic or 3−epicholic acid
oxocholic acid analog
Secondary Conjugated
glycolithocholic acid
glycoursodeoxycholic acid
glycodehydrocholic acid
glycohyodeoxycholic acid
tauroursodeoxycholic acid
taurohyodeoxycholic acid
glycodeoxycholic acid
taurolithocholic acid
taurodeoxycholic acid
Ctr
GV 100mg/kg
Ctr
GV 100mg/kg
Male
Female

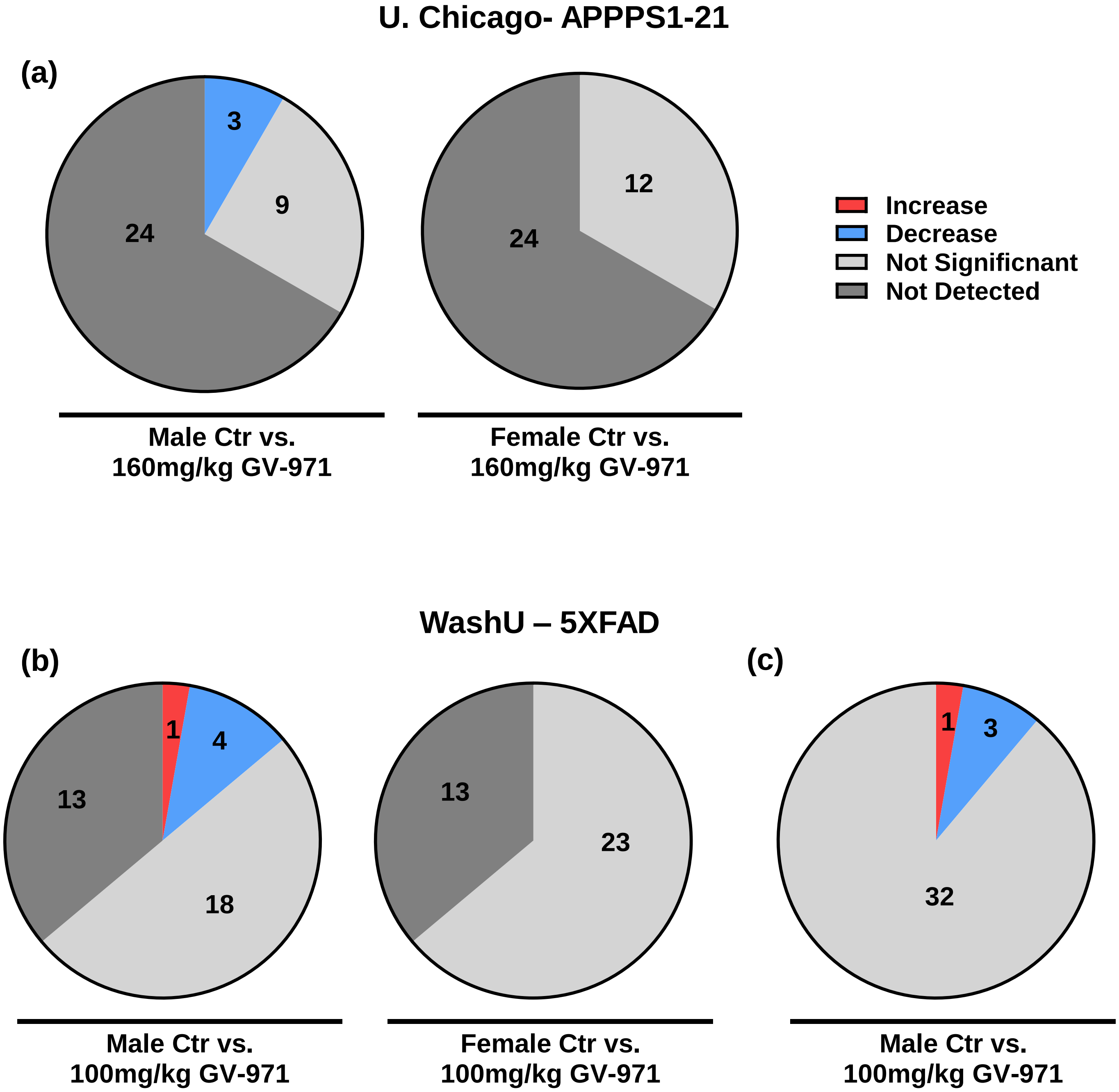
U. Chicago- APPPS1-21
(a)
3
9
24
12
24
Increase
Decrease
Not Significnant
Not Detected
Male Ctr vs.
160mg/kg GV-971
Female Ctr vs.
160mg/kg GV-971
WashU – 5XFAD
(b)
1
4
13
18
13
23
(c)
1
3
32
Male Ctr vs.
100mg/kg GV-971
Female Ctr vs.
100mg/kg GV-971
Male Ctr vs.
100mg/kg GV-971

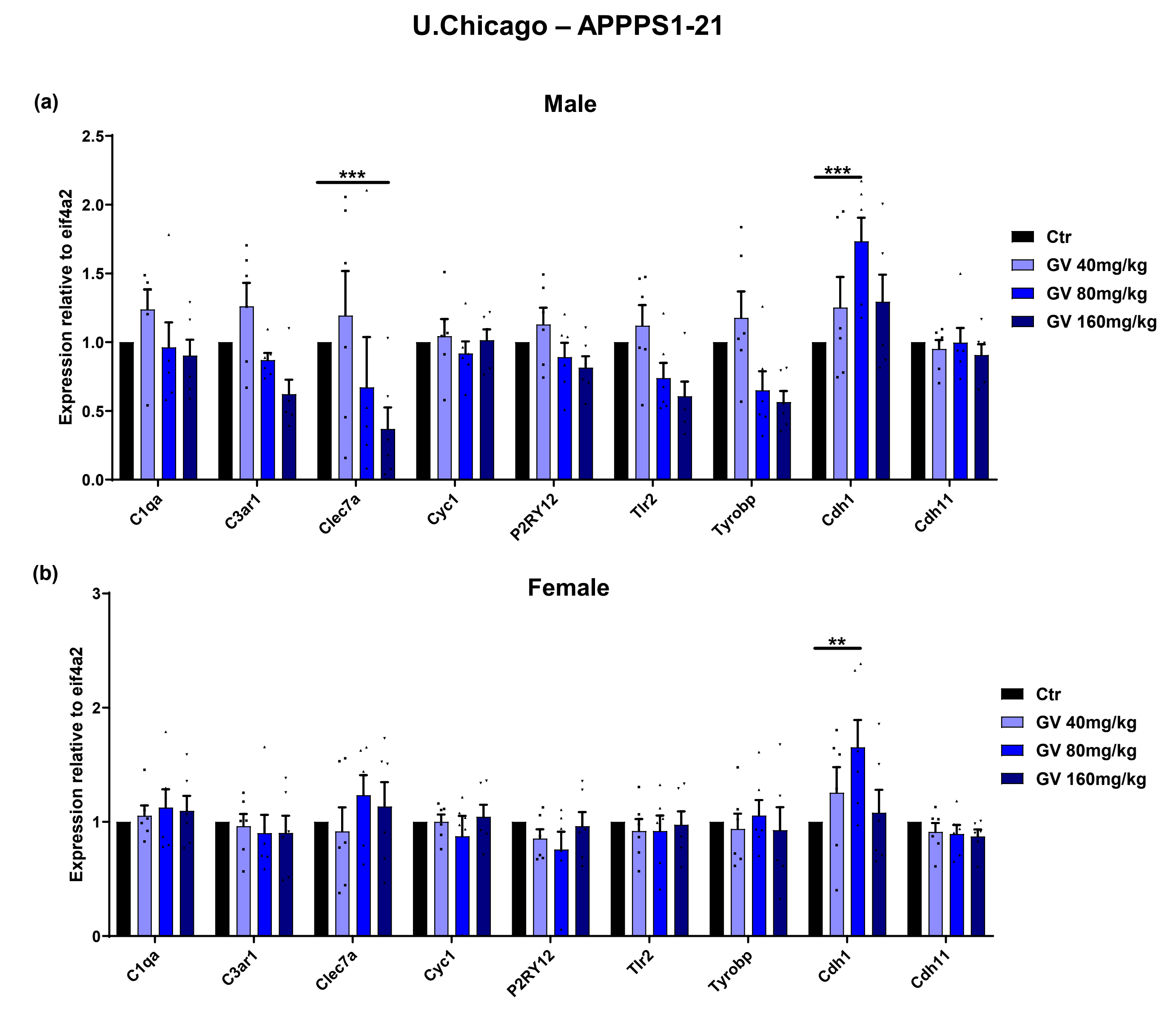
U.Chicago – APPPS1-21
(a)
Male
Expression relative to eif4a2
0.0
0.5
1.0
1.5
2.0
2.5
***
***
C1qa
C3ar1
Clec7a
Cyc1
P2RY12
Tlr2
Tyrobp
Cdh1
Cdh11
Ctr
GV 40mg/kg
GV 80mg/kg
GV 160mg/kg
(b)
Female
Expression relative to eif4a2
0
1
2
3
**
C1qa
C3ar1
Clec7a
Cyc1
P2RY12
Tlr2
Tyrobp
Cdh1
Cdh11
Ctr
GV 40mg/kg
GV 80mg/kg
GV 160mg/kg

WashU- 5XFAD

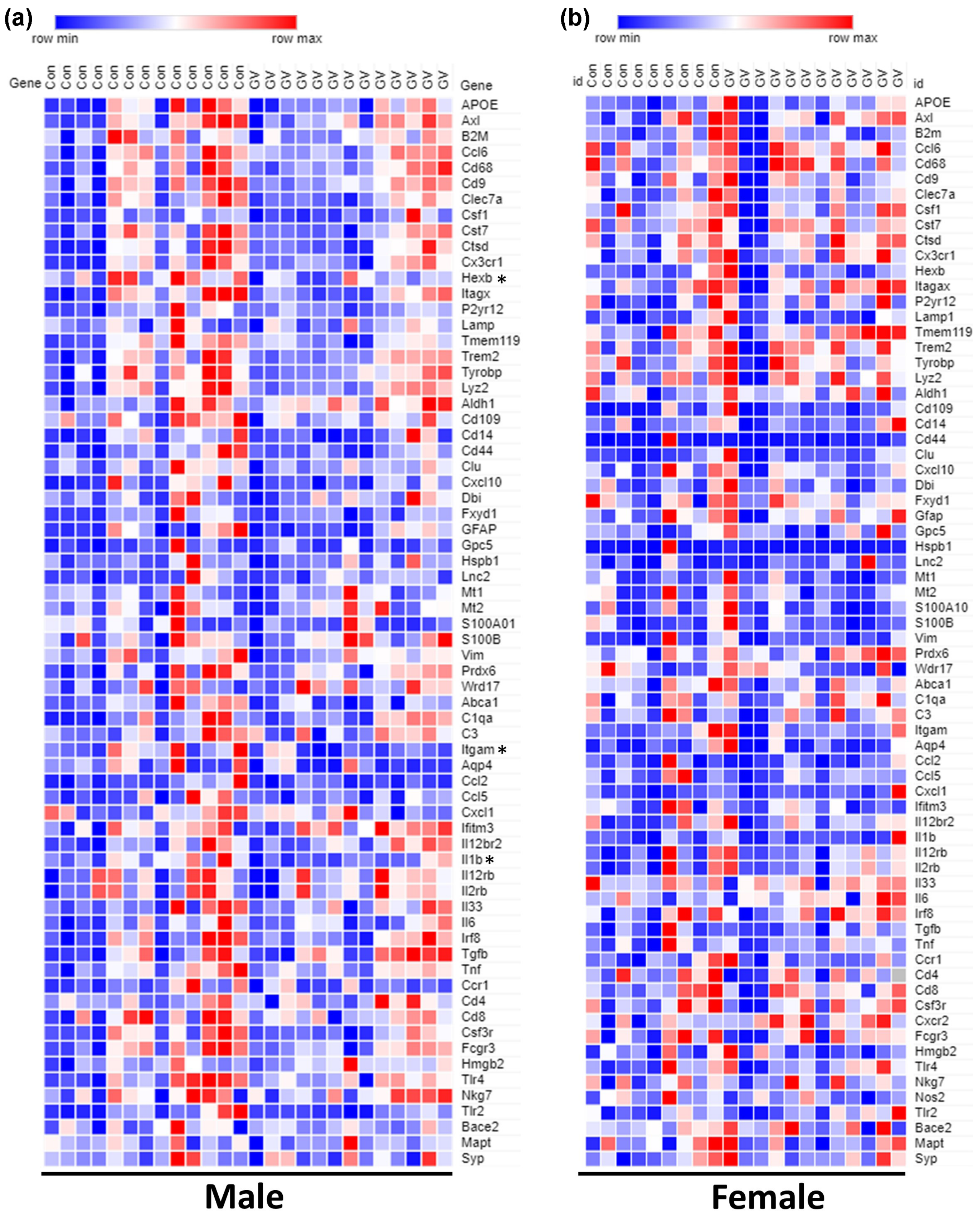
(a)
row min
row max
Gene
Con
GV
APOE
Axl
B2M
Ccl6
Cd68
Cd9
Clec7a
Csf1
Cst7
Ctsd
Cx3cr1
Hexb *
Itagx
P2yr12
Lamp
Tmem119
Trem2
Tyrobp
Lyz2
Aldh1
Cd109
Cd14
Cd44
Clu
Cxcl10
Dbi
Fxyd1
GFAP
Gpc5
Hspb1
Lnc2
Mt1
Mt2
S100A01
S100B
Vim
Prdx6
Wrd17
Abca1
C1qa
C3
Itgam *
Aqp4
Ccl2
Ccl5
Cxcl1
Ifitm3
Il12br2
Il1b *
Il12rb
Il2rb
Il33
Il6
Irf8
Tgfb
Tnf
Ccr1
Cd4
Cd8
Csf3r
Fcgr3
Hmgb2
Tlr4
Nkg7
Tlr2
Bace2
Mapt
Syp
Male
(b)
row min
row max
id
Con
GV
APOE
Axl
B2m
Ccl6
Cd68
Cd9
Clec7a
Csf1
Cst7
Ctsd
Cx3cr1
Hexb
Itagax
P2yr12
Lamp1
Tmem119
Trem2
Tyrobp
Lyz2
Aldh1
Cd109
Cd14
Cd44
Clu
Cxcl10
Dbi
Fxyd1
Gfap
Gpc5
Hspb1
Lnc2
Mt1
Mt2
S100A10
S100B
Vim
Prdx6
Wdr17
Abca1
C1qa
C3
Itgam
Aqp4
Ccl2
Ccl5
Cxcl1
Ifitm3
Il12br2
Il1b
Il12rb
Il2rb
Il33
Il6
Irf8
Tgfb
Tnf
Ccr1
Cd4
Cd8
Csf3r
Cxcr2
Fcgr3
Hmgb2
Tlr4
Nkg7
Nos2
Tlr2
Bace2
Mapt
Syp
Female
