# Supplementary material for: Sodium oligomannate alters gut microbiota, reduces cerebral amyloidosis and reactive microglia in a sex-specific manner
Source: Mol Neurodegener. 2024 Feb 17;19:18. doi: 10.1186/s13024-023-00700-w (PMC10874048; doi:10.1186/s13024-023-00700-w)
Supplement: Supplementary file 1 — Additional file 1: Supplemental Figure 1. Gut microbiome composition differs significantly between University of Chicago and Washington University in St. Louis. Analysis of bacterial α-diversity and β-diversity in fecal content from 9-week-old APPPS1-21 male mice collected at the University of Chicago and Washington University in St. Louis. (a) Shannon index, (b) Pielou species evenness. (d) PCoA plot generated by using unweighted unifrac distance metric. Diversity analyses, including alpha and beta diversity, alpha rarefaction, and group significance were analyzed by QIIME and QIIME2. Data are presented as mean SEM. Significance was determined using Two-way ANOVA . *, P < 0.05; **, P < 0.01; ***, P < 0.001; ****, P < 0.0001. Supplemental Figure 2. GV-971 targets Aβ plaque halo in a sex-dependent manner. (a) Representative immunofluorescent images of HJ3.4+ Aβ (red) surrounding X34+ Aβ (blue). White * indicates regions of reduced plaque halo. (b,c) Quantification of an average number of HJ3.4 + Aβ surfaces within 5μM X34+ Aβ surface plaque in cortices of 5XFAD mice treated with 100mg/kg GV-971 or vehicle (male = 13, female = 9-12). Data are presented as mean SEM. Significance was determined using unpaired t-test (d). *, P < 0.05; **, P < 0.01; ***, P < 0.001; ****, P < 0.0001. Supplemental Figure 3. GV-971 alters amino acid metabolism. GC-nCI-MS and PFBBR derivtization heatmap analysis of metabolite abundance in cecal content from 5XFAD mice treated with 100mg/kg GV-971 or vehicle (male = 13, female 9-12). Supplemental Figure 4. GV-971 modifies tryptophan metabolism. LCMS/MS heatmap analysis of tryptophan pathway, indole pathway, and kynurenine pathway metabolite concentrations in cecal content from 5XFAD mice treated with 100mg/kg GV-971 or vehicle (male = 13, female 9-12). Supplemental Figure 5. GV-971 influences primary and secondary bile acid metabolism. LCMS/MS heatmap analysis of primary and secondary bile acid concentrations in cecal content from 5XFAD mice t [file 13024_2023_700_MOESM1_ESM.zip › GV-971 Supplemental Revision.docx]

**Supplemental Materials**

**MATERIAL AND METHODS**

**Aβ plaque morphology characterization:** Immunofluorescence slides were stained with markers for X34, fibrillary plaques (Alexa 405) and HJ3.4 pan Aβ plaques (Alexa 647) and scanned using Leica SP5 laser scanning confocal microscope under 20x objective. Three 3D-Z stacks, 2μm step increments in the z-plane, were acquired from cortical tissues, 3 images per mouse. Quantification of confocal images for HJ3.4/X34 was performed on a semi-automated platform using MATLAB and Imaris 9.3.1 software to create surfaces of each stain. The X34 plaque surfaces were then extended 5μM around and the number of counter stain surfaces within the plaque perimeter was quantified and plotted using GraphPad Prism (Prism 9.4.1)

**Reverse Transcription Quantitative PCR:** We used 1 μg of RNA for cDNA synthesis using the SuperScript IV VILO Master Mix with exDNase Enzyme (cat #11766050, ThermoFisher) according to the kit instructions. After the synthesis, cDNA was diluted 40 times in Nuclease-free water (cat # AM9938, Invitrogen). Primers used for detection of signal were designed either by using established ones in previously published methods or using Primer BLAST tool (NCBI distribution) and checked for dimer formation in Multiple Primer Analyzer software (ThermoFisher). Please see the Supplemental Table 1 for the information of primers used. Supplemental figure 1 shows an image of the primer evaluation. cDNA were subject to qPCR using PowerUp SYBR Green Master Mix (cat # A25742, Applied Biosystems by Life Technologies). The volume of each reaction was 10 𝜇L, which contained 5𝜇L of PowerUP SYBR Green Master Mix (2x), 0.5 𝜇L of forward and reverse primer mixture (in concentration of 5 𝜇M), 0.4 𝜇L of Nuclease-free water, and 4 𝜇L of cDNA added to wells individually. The reactions were run on MicroAmp Fast Optical 96-Well Reaction Plate with Barcode (0.1 mL, cat # 4346906, Applied Biosystems by Life Technologies) with MicroAmp Optical Adhesive Film (cat # 4311971, Applied Biosystems by Life Technologies) on QuantStudio 3 Real-Time PCR System (cat # A28567, ThermoFisher). Amplification was performed starting with 3 minutes hold at 95 °C to activate the enzyme. Next, the template was denaturated at 95 °C for 20 seconds, then annealed at 60 °C for 20 seconds, which was followed by extension and data acquisition at 72 °C for 20 seconds. The analysis was performed as described (Annese et al. 2018). In brief, the data was normalized by evaluation of Ct mean of housekeeping gene (Cyc1) for each sample. Cyc1 was the most stable housekeeping gene, hence only this gene was included in analysis. Then the expression levels were calculated according to the ΔΔCt method. To assess the statistical differences between gene expressions Two-way ANOVA was performed. A p-value of <0.05 was considered statistically significant.

**Supplemental Figure 1.** **Gut microbiome composition differs significantly between University of Chicago and Washington University in St. Louis.** Analysis of bacterial α-diversity and β-diversity in fecal content from 9-week-old APPPS1-21 male mice collected at the University of Chicago and Washington University in St. Louis. **(a)** Shannon index, **(b)** Pielou species evenness. **(d)** PCoA plot generated by using unweighted unifrac distance metric. Diversity analyses, including alpha and beta diversity, alpha rarefaction, and group significance were analyzed by QIIME and QIIME2. Data are presented as mean SEM. Significance was determined using Two-way ANOVA . *, P < 0.05; **, P < 0.01; ***, P < 0.001; ****, P < 0.0001.

**Supplemental Figure 2. GV-971 targets Aβ plaque halo in a sex-dependent manner.** **(a)** Representative immunofluorescent images of HJ3.4^+^ Aβ (red) surrounding X34^+^ Aβ (blue). White * indicates regions of reduced plaque halo. **(b,c)** Quantification of an average number of HJ3.4 ^+^ Aβ surfaces within 5μM X34^+^ Aβ surface plaque in cortices of 5XFAD mice treated with 100mg/kg GV-971 or vehicle (male = 13, female = 9-12). Data are presented as mean SEM. Significance was determined using unpaired t-test (d). *, P < 0.05; **, P < 0.01; ***, P < 0.001; ****, P < 0.0001.

**Supplemental Figure 3. GV-971 alters amino acid metabolism.** GC-nCI-MS and PFBBR derivtization heatmap analysis of metabolite abundance in cecal content from 5XFAD mice treated with 100mg/kg GV-971 or vehicle (male = 13, female 9-12).

**Supplemental Figure 4.** **GV-971 modifies tryptophan metabolism.** LCMS/MS heatmap analysis of tryptophan pathway, indole pathway, and kynurenine pathway metabolite concentrations in cecal content from 5XFAD mice treated with 100mg/kg GV-971 or vehicle (male = 13, female 9-12).

**Supplemental Figure 5. GV-971 influences primary and secondary bile acid metabolism.** LCMS/MS heatmap analysis of primary and secondary bile acid concentrations in cecal content from 5XFAD mice treated with 100mg/kg GV-971 or vehicle (male = 13, female 9-12).

**Supplemental Figure 6. GV-971 Significantly change peripheral and neuro-cytokine and chemokine production.** Pie chart denoting the distribution of cytokine/chemokine production following GV-971 treatment. Chart is characterized into four groups based on expression levels compared to the control groups: Increased, Decreased, Not significant, and Non Detected. **(a).** Serum analyzed from the University of Chicago APPPS1-21 male and female mice treated with 160mg/kg GV-971. **(b).** Serum analyzed from Washington University 5XFAD male and female mice treated with 100mg/kg GV-971. **(c).** Cortical tissue analyzed from Washington University 5XFAD male mice treated with 100mg/kg GV-97.

**Supplemental Figure 7. GV-971 significantly alters microglia activation and neurodevelopment gene expression. (a).** Quantitative PCR analysis of inflammatory, microglial, and neurodevelopment gene expression from bulk cortical tissue of male APPPS1-21 mice treated with GV-971 or vehicle**. (b)** Quantitative PCR analysis of inflammatory, microglial, and neurodevelopment gene expression from bulk cortical tissue of female APPPS1-21 mice treated with GV-971 or vehicle. Data are presented as mean SEM. Significance was determined using 2 way ANOVA followed by post hoc Tukey’s multiple comparisons. *, P < 0.05; **, P < 0.01; ***, P < 0.001; ****, P < 0.0001.

**Supplemental Figure 8**. **GV-971 alters inflammatory markers in 9 month old 5XFAD male mice.** Heat map analysis of bulk RNA in cortices of 5XFAD mice following 100mg/kg GV-971 or vehicle treatment **(A)** male mice n=13, **(B)** female mice n= 9-12. Graph generated by hierarchical gene clustering based on groups. Statistical analyses were performed using an unpaired t- test. *, P < 0.05
